# Associations between Meteorological Parameters and Influenza Activity in Berlin (Germany), Ljubljana (Slovenia), Castile and León (Spain) and Israeli Districts

**DOI:** 10.1371/journal.pone.0134701

**Published:** 2015-08-26

**Authors:** Radina P. Soebiyanto, Diane Gross, Pernille Jorgensen, Silke Buda, Michal Bromberg, Zalman Kaufman, Katarina Prosenc, Maja Socan, Tomás Vega Alonso, Marc-Alain Widdowson, Richard K. Kiang

**Affiliations:** 1 Goddard Earth Sciences Technology and Research, Universities Space Research Associations, Columbia, Maryland, United States of America; 2 Global Change Data Center, NASA Goddard Space Flight Center, Greenbelt, Maryland, United States of America; 3 Regional Office for Europe, World Health Organization, Copenhagen, Denmark; 4 Influenza Division, U.S. Centers for Disease Control and Prevention (CDC), Atlanta, Georgia, United States of America; 5 Robert Koch Institute, Berlin, Germany; 6 Israel Center for Disease Control, Ministry of Health, Tel-Hashomer, Israel; 7 Laboratory for Virology, National Institute of Public Health Slovenia, Ljubljana, Slovenia; 8 Communicable Diseases and Environmental Health Care, National Institute of Public Health, Ljubljana, Slovenia; 9 Public Health Directorate, Health Department, Valladolid, Spain; Columbia University, UNITED STATES

## Abstract

**Background:**

Studies in the literature have indicated that the timing of seasonal influenza epidemic varies across latitude, suggesting the involvement of meteorological and environmental conditions in the transmission of influenza. In this study, we investigated the link between meteorological parameters and influenza activity in 9 sub-national areas with temperate and subtropical climates: Berlin (Germany), Ljubljana (Slovenia), Castile and León (Spain) and all 6 districts in Israel.

**Methods:**

We estimated weekly influenza-associated influenza-like-illness (ILI) or Acute Respiratory Infection (ARI) incidence to represent influenza activity using data from each country’s sentinel surveillance during 2000–2011 (Spain) and 2006–2011 (all others). Meteorological data was obtained from ground stations, satellite and assimilated data. Two generalized additive models (GAM) were developed, with one using specific humidity as a covariate and another using minimum temperature. Precipitation and solar radiation were included as additional covariates in both models. The models were adjusted for previous weeks’ influenza activity, and were trained separately for each study location.

**Results:**

Influenza activity was inversely associated (p<0.05) with specific humidity in all locations. Minimum temperature was inversely associated with influenza in all 3 temperate locations, but not in all subtropical locations. Inverse associations between influenza and solar radiation were found in most locations. Associations with precipitation were location-dependent and inconclusive. We used the models to estimate influenza activity a week ahead for the 2010/2011 period which was not used in training the models. With exception of Ljubljana and Israel’s Haifa District, the models could closely follow the observed data especially during the start and the end of epidemic period. In these locations, correlation coefficients between the observed and estimated ranged between 0.55 to 0.91and the model-estimated influenza peaks were within 3 weeks from the observations.

**Conclusion:**

Our study demonstrated the significant link between specific humidity and influenza activity across temperate and subtropical climates, and that inclusion of meteorological parameters in the surveillance system may further our understanding of influenza transmission patterns.

## Introduction

Influenza is an acute respiratory infection that continues to be a serious global public health and economic concern. WHO estimates that seasonal influenza can result in up to 500,000 deaths and 5 million severe illnesses worldwide annually [1]. In Europe, there could be around 38,500 excess deaths due to influenza (out of ~500 million population) but with considerable variations each year [2]. The latest pandemic due to A(H1N1)pdm09 was estimated to cause 280,000 deaths, with most among the non-elderly adults, in contrast to seasonal influenza where mortality is highest in the elderly [3].

The timing of seasonal influenza epidemic varies across latitude, suggesting the involvement of meteorological and environmental conditions. In temperate regions (23°27’−66°33’N and 23°27’−66°33’S), influenza epidemics typically occur during winter time—December to March in the Northern Hemisphere and May to September in the Southern Hemisphere [4]. In the tropics (23°27’N−23°27’S) and in some locations in the subtropics (23°27’−35°N and 23°27’−35°S), however, influenza seasonality is less clear and the epidemic pattern varies widely: from year-round high influenza activity, peaks that coincide with rainy seasons, to multiple peaks in a year [5–8].

Influenza transmission generally increases under conditions and settings that promote [9]: influenza virus survival, or more frequent contacts with infected humans or contaminated objects, and when there is insufficient human immunity against circulating influenza virus. Meteorological and environmental conditions may therefore influence how easily infection may take place. Winter time meteorological conditions (temperature, humidity and solar radiation) are often associated with influenza epidemics in the temperate regions [10–12]. Such favorable conditions were corroborated through laboratory and animal studies: low temperature and humidity increased transmission efficiency [13,14] and prolonged virus survival outside the body [15,16]. The contribution of solar radiation on influenza seasonality in the temperate region, however, remains inconclusive [17]. Solar radiation’s ultraviolet may (i) stimulate vitamin D production, modulate the immune system and safeguard the body against influenza virus [18]; and (ii) inactivate influenza viruses [19], though excessive UV exposure may also damage immune system. Influenza in the tropics, on the other hand, is more frequently associated with rainfall though the direct causal link remains to be established. It is postulated that rainfall can also promote indoor crowding that increases the probability of both aerosol and contact transmission.

In Europe, association with absolute humidity has been demonstrated at national level in several countries [20,21]. These studies showed inverse relationship with absolute humidity which accounted for up to 3% of influenza variability. A study in Israel [22] indicated that both temperature and humidity modulated the country’s influenza transmission. Furthermore, another study demonstrated through a mathematical model and forecast that influenza dynamics in Israel was influenced by a combination of temperature, humidity, antigenic drift and immunity lost [23].

**Fig 1:**
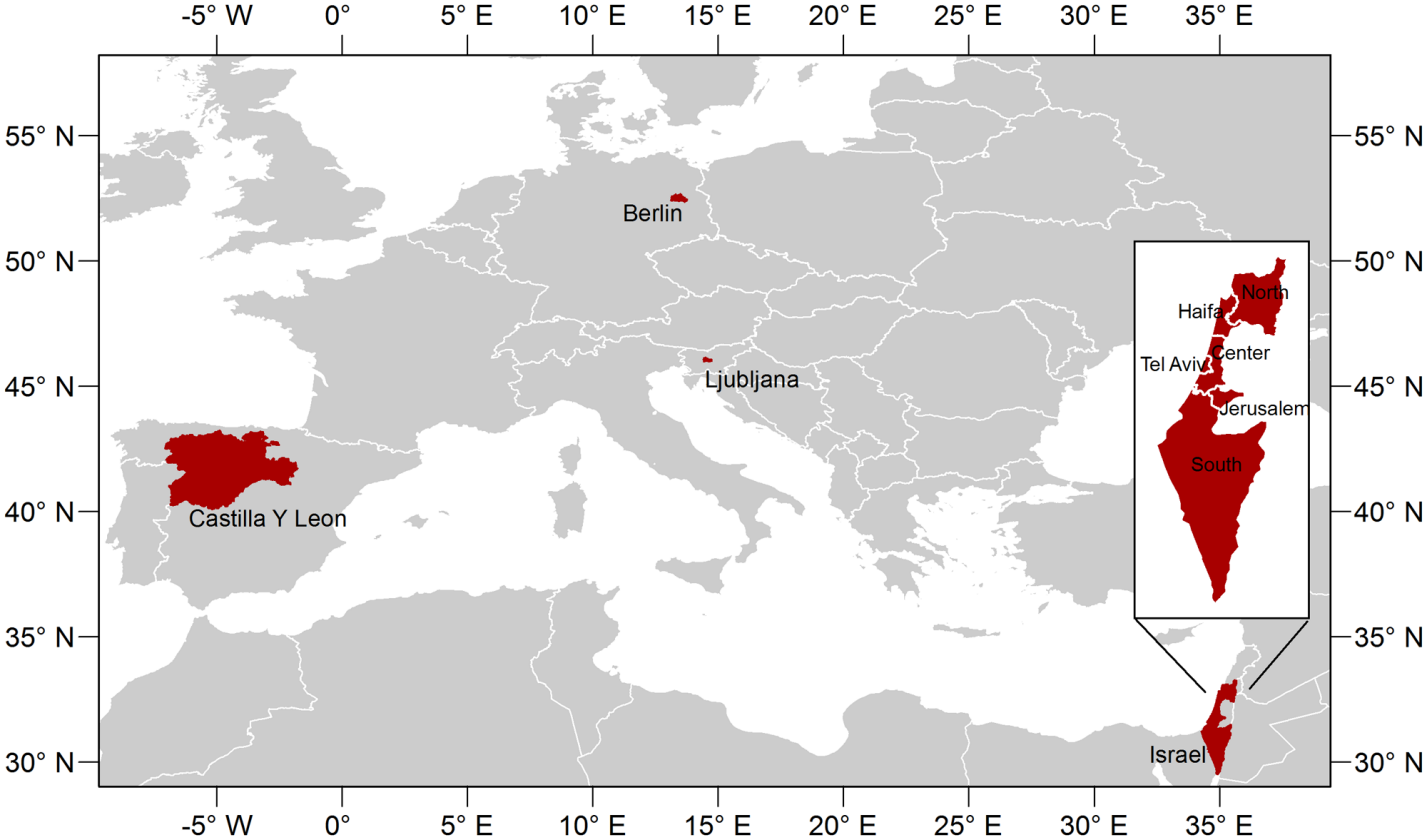
Study Locations.

Because there had been varying associations between meteorological parameters and influenza activity across climate zones, we investigated such relationship in two different climates (Fig 1)–Germany, Slovenia and Spain in the temperate zone, and Israel in the subtropical zone. Furthermore, since temperature, precipitation, humidity and other parameters may vary significantly within a climatic zone and even a country, our study was conducted at sub-national level (Fig 1). We used 9 sets of sub-national influenza data ranging from township (Ljubljana in Slovenia), city (Berlin in Germany), district (all 6 districts in Israel: North, Haifa, Tel Aviv, Center, Jerusalem and South) to community (Castile and León in Spain).

## Materials and Methods

### Influenza Data

Influenza sentinel surveillance data was collected from 2000 to 2011 for Castile and León (Spain), and 2006–2011 for all the other locations. The German influenza sentinel system is based on Acute Respiratory Infection (ARI) surveillance, while the systems in other countries are based on Influenza-Like-Illness (ILI) surveillance. For each study location, we obtained the weekly number of: (i) ILI or ARI cases, (ii) respiratory samples tested for influenza, (iii) respiratory samples tested positive for influenza, and (iv) the population covered by the sentinel system. Respiratory samples were tested by RT-PCR with the date of the visit to the physicians recorded. Detailed description of each country’s sentinel surveillance system can be found in S1 Text.

In order to obtain indicators for influenza activity, we multiplied the number of weekly ILI or ARI cases by the proportion of respiratory samples that were tested positive for influenza from the corresponding week. We then divided this number by the population served by the practitioners in that sentinel surveillance area to obtain the rate of medically attended influenza-associated ILI or ARI cases per 100,000 population. The areas and populations for the study locations are given in Table 1.

**Table 1:** Study locations and influenza data summary.

Country	Sub-National Locations	Area (km^2^)	Population	Study Period	Total Specimens Tested*	% Positive for Influenza*
**Germany**	Berlin	891	3,520,061	2006–2011	2258	47.3
**Slovenia**	Ljubljana	164	280,278	2006–2011	319	31.66
**Spain**	Castilla y León	94,222	2,558,463	2000–2011	1255	37.05
**Israel**	Northern District	3,320	1,241,900	2006–2011	416	29.33
	Haifa District	866	880,700	2006–2011	162	30.25
	Central District	1,294	1,770,000	2006–2011	1461	30.39
	Tel Aviv District	172	1,227,900	2006–2011	582	42.1
	Jerusalem District	653	907,300	2006–2011	1049	42.33
	Southern District	14,185	1,201,200	2006–2011	935	28.77

* Excluding observations during pandemic period (May 2009–May 2010).

### Meteorological Data

In this study, we assessed the relationship between influenza activity and 4 meteorological parameters that have been implicated in influenza transmission as described previously: specific humidity, minimum temperature, precipitation and solar radiation. Specific humidity measures the mass of water vapor in a unit mass of air (expressed in g/kg). Unlike relative humidity, it does not depend on temperature and is conceptually similar to absolute humidity, which measures the mass of water vapor in a unit volume of air (expressed in g/m^3^). Hence, the dynamics of specific humidity is similar to that of absolute humidity. Several influenza studies have used specific humidity as a measure of absolute humidity [10,24]. For air temperature, there are typically 3 measurements—minimum, mean and maximum temperature. An animal study has indicated that influenza transmission was associated with cold temperature [13]. Hence is the use of minimum temperature in this study, although those three measures were indeed strongly correlated with one another.

Daily minimum temperature was obtained from ground station data archived at the National Climatic Data Center (NCDC) [25] (for Spain, Slovenia and Israel) and at Germany’s climate data center [26] (for Berlin). Daily precipitation for Spain, Slovenia and Israel were obtained from the Tropical Rainfall Measuring Mission (TRMM) satellite via NASA’s Interactive Online Visualization And Analysis Infrastructure (GIOVANNI) [27]. Specific humidity and solar radiation for all study locations, as well as precipitation for Berlin, were obtained from the Global Land Data Assimilation System (GLDAS)[28]–a system that utilizes ground and satellite measurements to model global terrestrial geophysical parameters with contiguous spatial and temporal coverage. Both TRMM and GLDAS datasets have 0.25° spatial resolution (~25 km at the equator), and they are available for download from NASA Goddard Earth Sciences Data and Information Services Center (GES DISC) GIOVANNI portal [29].

When there was more than one ground station, we first averaged the daily data across stations before calculating the weekly average. For TRMM and GLDAS data, we averaged all pixels that had more than 10% of its pixel coverage within the study region, and subsequently took the weekly average. Normally influenza incubation period is 1–4 days and infectious period (when viral shedding occurs) is up to 7 days (10 days or more in children). A number of studies however have shown that influenza activity can be associated with meteorological conditions up to 2 months earlier [9,10,12].

### Analytic Approach

We employed Generalized Additive Model (GAM) [30] which can account for nonlinear relationship by using smoothing splines function of the covariates. GAM is commonly used in mortality-pollution studies [31–33] and was used to relate meteorological factors to influenza-associated mortality [34]. Covariates included in the models were the meteorological parameters and the previous week’s influenza activity (to account for autocorrelation in the time series). Due to high correlation between specific humidity (SH) and minimum temperature (TMIN) (correlation coefficient 0.86–0.98), we developed two models—one with SH and another with TMIN—such that the two variables did not enter the model concurrently in order to avoid spurious relationship due to concurvity. For each location *k*, the two models are: Model 1 (SH):

$$\text{ln}(y_{t,k})\;=\;\alpha +\;s(SH_{t_{1},k})+\;s(P_{t_{1},k})+\;s(SRAD_{t_{1},k})\;+\;s(\text{ln}(y_{t_{1},k}))\;$$ 1

Model 2 (TMIN):

$$\text{ln}(y_{t,k})\;=\;\alpha +\;s(TMIN_{t_{1},k})+\;s(P_{t_{1},k})+\;s(SRAD_{t_{1},k})\;+\;s(\text{ln}(y_{t_{1},k}))\;$$ 2

where *y*_*t*,*k*_ is the influenza-associated ILI or ARI at time *t* and location *k*; *α* is the intercept; *s(·)* indicates smooth spline function, in particular we used penalized cubic regression smoothing splines [35] (S1 Text); *SH* is specific humidity (in g/kg); *P* is precipitation (mm); and *SRAD* is solar radiation (W/m^2^). Subscript *t*_*1*_ indicates 1-week lag. The model assumed over dispersed Poisson structure [35]. Population was entered as an offset such that the regression was for rate of influenza-associated ILI or ARI per population, which is referred as influenza activity henceforth. The models were fitted for each study location separately. All analysis was performed using R software [36] with the mgcv package for GAM. Model estimation was performed using Penalized Iteratively Reweighted Least Squares (P-IRLS) [35].

We applied backward selection for the meteorological variables, with the model performance as selection criteria. The model performance was measured by Generalized Cross Validation (GCV) score [35], which is essentially the model’s error that also accounts for the degrees of freedom (see S1 Text). In fitting the model, we inflated the GCV score (setting gamma = 1.4) in order to reduce over-fitting without degrading much of the prediction error performance as suggested by Wood [35]. Autocorrelation was subsequently assessed using autocorrelation and partial autocorrelation function graphs. If significant partial autocorrelation was present in the first two lags [37], we increased the splines’ maximum allowable degree of freedom by 1, to a maximum of 6. If autocorrelation persisted, we added the 2-week lag of the dependent variables. The significance of each smooth term in the model was tested (smooth function, s(.) = 0) using Bayesian confidence intervals for the smooths as described in Wood [38]. The shapes of the resulting meteorological smooth terms were plotted with its 95% confidence limits (calculated using Bayesian confidence intervals [35]). A downward slope indicated an inverse relationship with the dependent variable, and an upward slope indicated a proportional relationship.

For each study location we divided the data into training and validation datasets. Validation datasets consisted of data from the last season (2010/2011). We excluded data during the pandemic year (May 2009-May 2010) from the analysis, because they may not well represent the typical influenza epidemics. The resulting model was used to estimate influenza activity for 2010/2011 season. We identified the peak week in this season by simply taking the maximum value, and calculated the differences between the time that the estimated and observed peak occur.

Using the data during an influenza season (taken as January-March), we calculated for each study location, the change in influenza activity when the significant meteorological covariates were increased, one at a time, by a small increment (10% of the range) from its median value, while the other covariates were held at their median values. These increments, on average across locations, corresponded to approximately 0.5g/kg for SH, 8.1 mm for precipitation, 16.4 W/m^2^ for SRAD and 0.6°C for TMIN. The associated change in influenza activity was expressed as percentage change relative to the influenza activity value when the meteorological parameters were at their median values. This analysis was performed to quantitatively assess how influenza varied with small changes in the meteorological parameter. We further assessed the contribution of the meteorological parameters to the model by removing one meteorological parameter at a time and calculated the percent change in the deviance from the full model (see S1 Text).

## Results

### Specific Humidity Model (Model 1)

**Fig 2:**
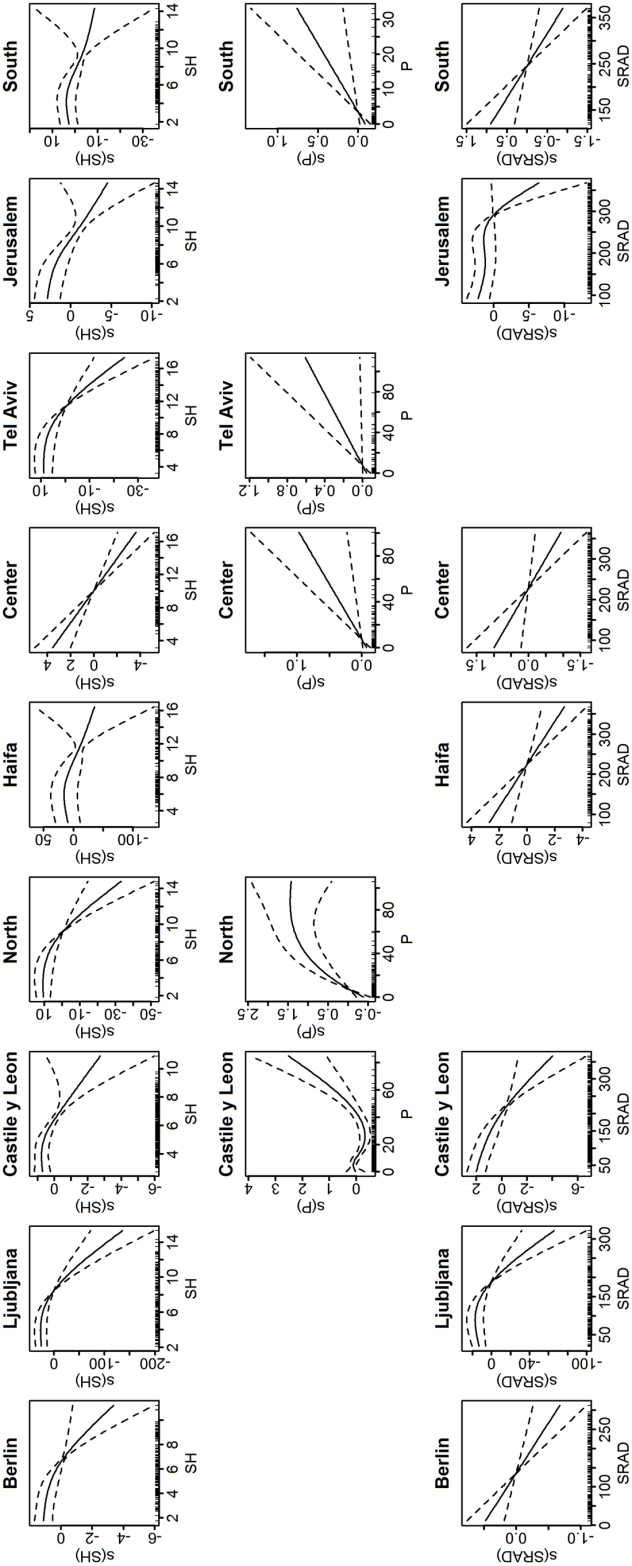
Plots of the meteorological smooth terms for Model 1 (with specific humidity). Only terms that are significant are plotted. The y-axis is in the predictor scale (log(y)) and normalized, while the x-axis is the value of the meteorological variable. The dashed lines are the 95% confidence interval. Downward slope indicates inverse relationship, while upward slope indicates proportional relationship.

Using GAM with backward selection applied to the meteorological covariates and adjusted for previous weeks’ influenza incidence, we found that influenza-associated ILI or ARI per 100,000 population (influenza activity) was inversely associated (p<0.05) with specific humidity (SH) in all locations (Table 2). Plots of the resulting SH smooth term (Fig 2) showed a decreasing trend, indicating an inverse association with influenza activity. In most locations, SH smooth plots showed log linear relationship with influenza activity. Such relationship was also revealed by the effective degree of freedoms (EDF in Table 2) of one, or approximately one. Association with precipitation was found in 1 temperate location and in 4 Israeli subtropics locations with higher EDF (1–2.52, Table 2) and varying trends (Fig 2): an increasing trend in Center, Tel Aviv and South districts of Israel, and both increasing and decreasing trends in Castile and León (Spain) and North district (Israel). Lastly, solar radiation (SRAD) was inversely associated with influenza activity in all 3 temperate locations and in more than half of the subtropics locations (Fig 2). Similar to SH, association with SRAD showed close-to-linear relationship (EDF 1–2.81). The models’ performance as measured by the adjusted R^2^ ranged from 0.19 to 0.75 (mean 0.59), with 51% to 86% deviance explained (Table 2).

**Table 2:** Model 1 (with specific humidity) regression parameters. Models were adjusted for previous weeks’ influenza activity.

	Meteorological Smooth Terms EDF (p-value)*			Adj.	% Dev.	Pred. Corr.
	Specific Humidity	Precipitation	Solar Radiation	R^2^	Explained	Coeff.^ǂ^
Berlin	1.72 (<0.001)		1 (0.001)	0.70	75.7	0.87
Ljubljana	1.96 (<0.001)	1.71 (0.06)	1.97 (<0.001)	0.34	63	0.02
Castile & León	1.91 (0.002)	2.52 (0.001)	1.61 (<0.001)	0.56	72.1	0.88
North	1.88 (<0.001)	1.81 (<0.001)	1 (0.07)	0.64	75.1	0.71
Haifa	1.91 (0.02)		1 (0.001)	0.19	51.2	0.33
Center	1 (<0.001)	1 (0.01)	1 (0.01)	0.71	83.2	0.86
Tel Aviv	1.88 (<0.001)	1 (0.04)		0.70	77.6	0.91
Jerusalem	1.78 (0.001)		2.81 (<0.001)	0.75	86.1	0.84
South	1.88 (0.002)	1 (0.009)	1 (0.003)	0.69	73.6	0.82

* EDF is the effective degree of freedom for the estimated smooth terms. Meteorological parameter units: g/kg for specific humidity, mm/day for precipitation, W/m^2^ for solar radiation.

^ǂ^ Correlation coefficient between the estimated influenza-associated ILI or ARI with the observed during 2010/2011 season.

When SH median value during influenza season was increased by 10% of the range (average of 0.5 g/kg across locations), influenza activity was decreased by 6.9–53.6% in all locations (Table 3). This percentage change indicated how much influenza activity changed when SH was increased, relative to the value of influenza activity at the time when SH was at its median value. Increase in precipitation (~ 8.1 mm) was associated with decreased influenza activity in Castile and León (18.7%) and increased influenza activity in North District (44.7%), Center District (11.04%), Tel Aviv District (6.2%) and South District (8.7%) (Table 3). Since the precipitation smooth term showed both decreasing and increasing trends (Fig 2), we further calculated the change in influenza activity from precipitation’s 90th percentile value and found that influenza increased by 21% (95% CI = 8.4–33.6%). The result for Castile and León indicated that higher amount of precipitation was proportionally associated with influenza activity while low precipitation had an inverse association. For SRAD, a small increase (~16.4 W/m^2^) from the median was associated with 4.5–27.2% decrease in influenza activity in Berlin, Castile and León, Haifa, Center, Jerusalem and South. There was no statistical changes in influenza activity in Ljubljana, but similar to precipitation, an increase from the 90^th^ percentile value was associated with decreased influenza activity by 94.3% (95% CI = 84.2–105.7%).

**Table 3:** Percentage change in influenza-associated ILI or ARI per 100,000 populations when one of the meteorological variables was increased from its median value by 10% of its range (Δx) during epidemic weeks (Model 1).

	Specific Humidity	Rainfall	Solar Radiation
Berlin	-6.89 (-11.19,-2.60)		-4.48 (-7.07,-1.89)
Ljubljana	-30.99 (-45.16,-16.81)		5.81 (-11.34,22.96)
Castile & Leon	-6.66 (-11.42,-1.89)	-18.73 (-28.62,-8.85)	-18.36 (-24.78,-11.95)
North	-53.61 (-66.47,-40.75)	44.74 (23.38,66.10)	
Haifa	-46.65 (-70.68,-22.62)		-27.17 (-40.49,-13.86)
Center	-22.02 (-30.55,-13.49)	11.04 (2.55,19.53)	-11.21 (-19.62,-2.79)
Tel Aviv	-31.51 (-40.17,-22.86)	6.18 (0.213,12.15)	
Jerusalem	-19.09 (-26.90,-11.27)		-8.41 (-14.30,-2.52)
South	-22.23 (-33.37,-11.09)	8.71 (1.95,15.47)	-10.64 (-16.98,-4.31)

**Fig 3:**
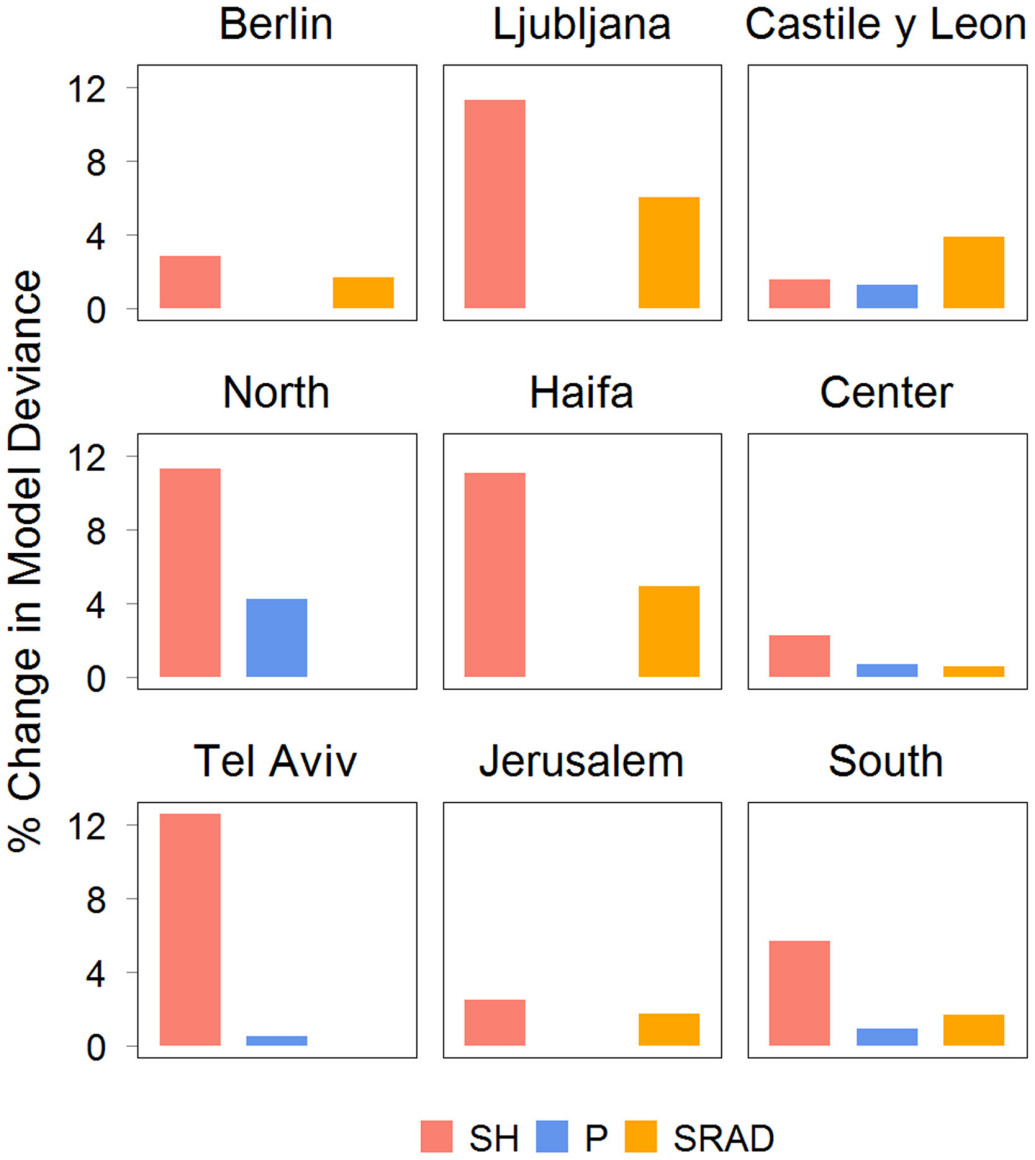
Percentage change in model deviance when the specified meteorological parameter was excluded from Model 2. SH is specific humidity, PRCP is precipitation and SR is solar radiation.

We estimated the contribution of the meteorological parameters to the model based on the changes in the model’s deviance when the specified meteorological covariate was removed (Fig 3). SH had the largest contribution (2.3%-11.3%) for all locations except in Castile and León. In Castile and León, SRAD had the largest contribution (3.91%).

**Fig 4:**
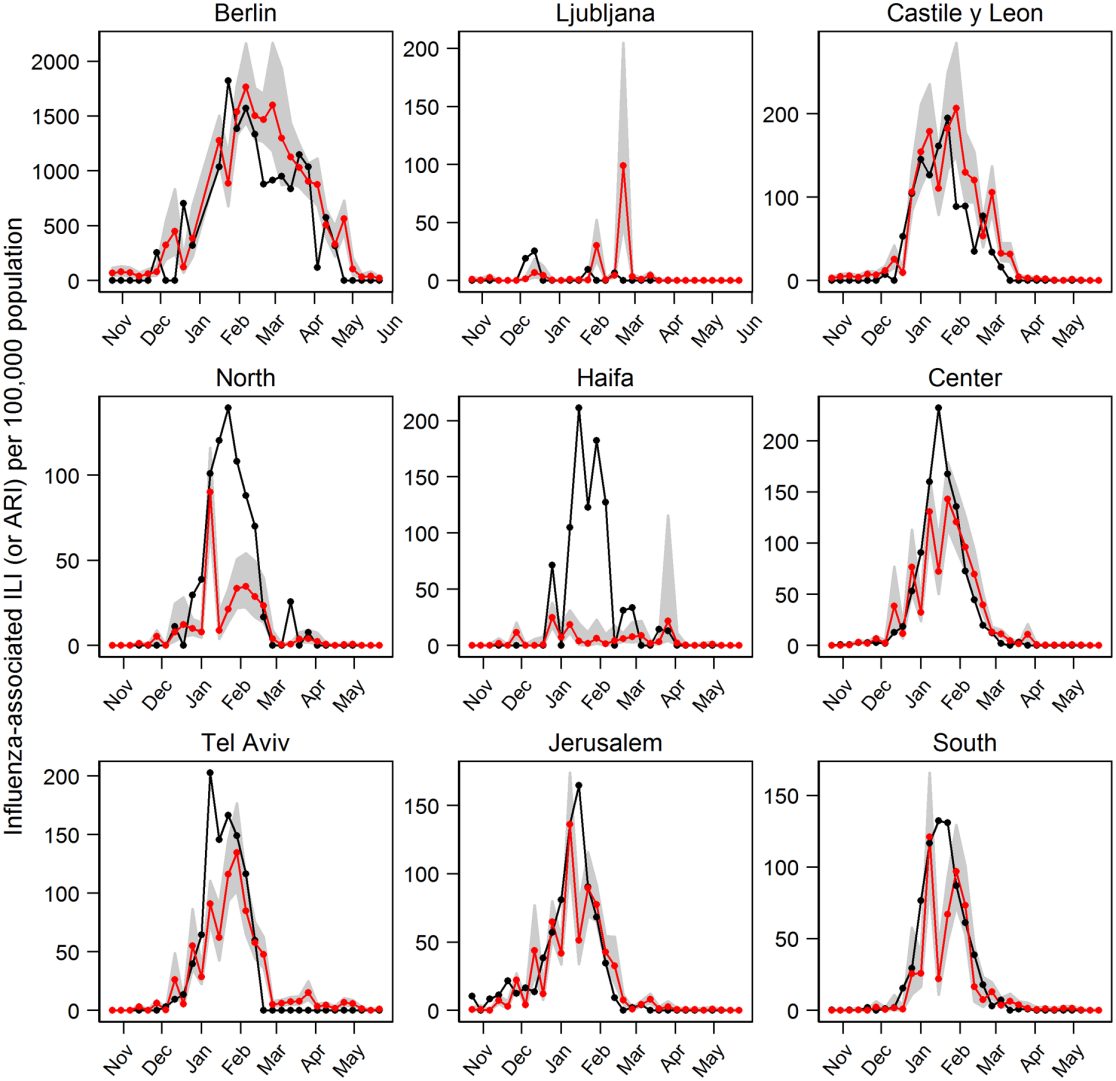
Estimated influenza activity in 2010/2011 season using Model 1 (with specific humidity). Black line is the observations, red line is the predicted influenza and the shaded areas are the 95% CI.

We further used the model to predict the influenza activity in the 2010/2011 season (Fig 4), which was not included in training the model (see S1 Fig for training data estimation). In 6 of the 9 study locations, the models closely followed the rise and fall of the curves. The models accurately estimated the peak week timing (defined simply as the maximum within the season) in Jerusalem and South; while for the other locations the peak week timing was estimated within 1 week (Berlin, Castile and León, Center and Haifa), 3 weeks (North and Tel Aviv) and 11 weeks (Ljubljana) of the observed. Correlation coefficients between the estimated and observed influenza activity (Table 2) were lowest in Ljubljana and Haifa (approximately 0.1), while in the other 7 locations it ranged from 0.74 to 0.95 (mean 0.88).

### Minimum Temperature Model (Model 2)

**Fig 5:**
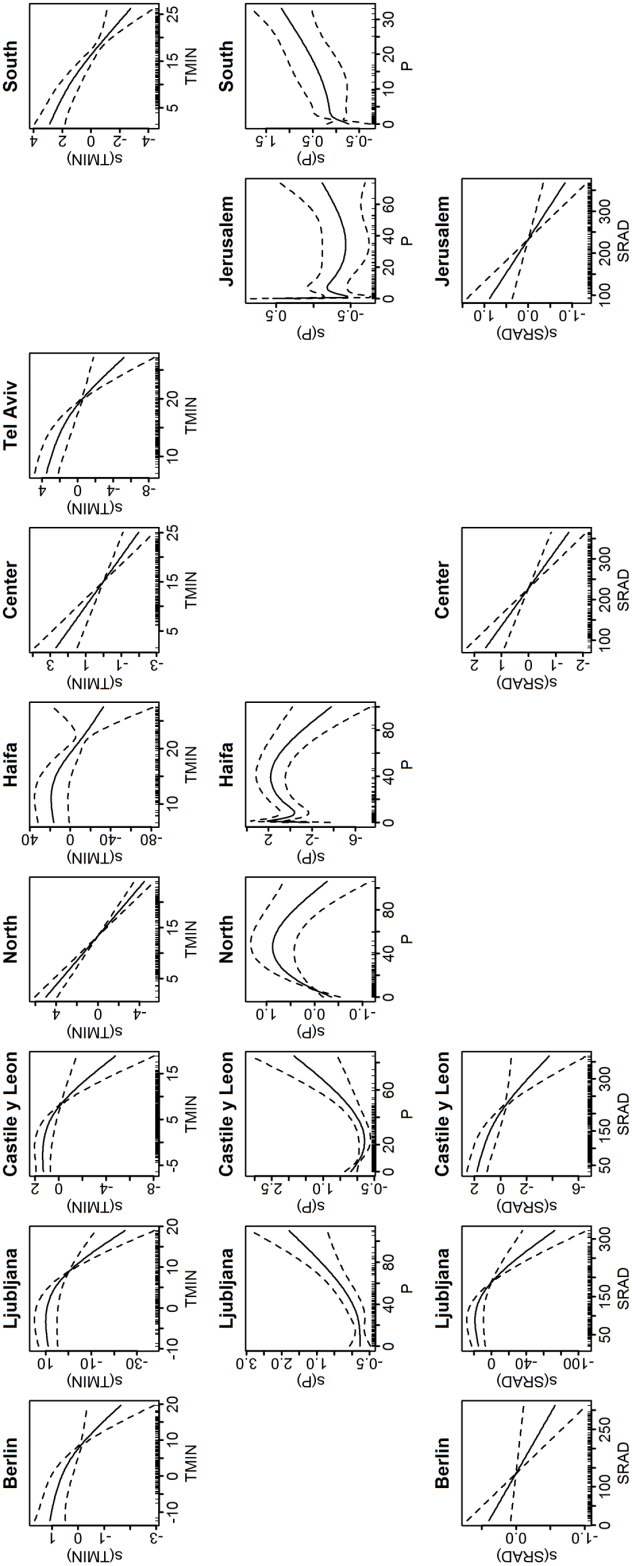
Plots of the meteorological smooth terms for Model 2 (with minimum temperature). Only terms that are significant are plotted. The y-axis is in the predictor scale (log(y)) and normalized, while the x-axis is the value of the meteorological variable. The dashed lines are the 95% confidence interval. Downward slope indicates inverse relationship, while upward slope indicates proportional relationship.

When minimum temperature (TMIN) was used as a covariate instead of SH, we found that TMIN was inversely associated (p<0.05) with influenza activity in all locations but Jerusalem District in Israel (Table 4). TMIN smooth terms’ EDF were, in general, higher than SH in Model 1 (EDF up to 2.82 in Table 4). Association with precipitation was observed in 6 out of the 9 locations. SRAD was associated with influenza activity in all temperate locations and in 2 of the 6 subtropics locations. Similar to Model 1, our results using Model 2 indicated: 1) a decreasing trend in influenza activity (log-scale) as TMIN increased (Fig 5); 2) a varying relationship between influenza activity and precipitation; and 3) a decreasing trend in influenza activity as SRAD increased (except in South where increasing trend was also observed). The adjusted R^2^ for Model 2 ranged from 0.30 to 0.72 with 61.8% to 85.4% deviance explained (Table 4).

**Table 4:** Model 2 (with minimum temperature) regression parameters. Models were adjusted for previous weeks’ influenza activity.

	Meteorological Smooth Terms EDF (p-value)*			Adj.	% Dev.	Pred. Corr.
	Min. Temp	Precipitation	Solar Radiation	R^2^	Explained	Coeff. ^ǂ^
Berlin	1.6 (0.006)		1 (0.01)	0.71	75.3	0.87
Ljubljana	1.95 (<0.001)	1.7 (0.008)	2 (<0.001)	0.30	61.8	0.10
Castile & León	1.84 (<0.001)	1.87 (0.007)	1.61 (<0.001)	0.56	72.0	0.84
North	1 (<0.001)	1.94 (<0.001)		0.58	74.5	0.55
Haifa	1.91 (0.003)	3.91 (<0.001)	2.98 (0.1)	0.54	65.8	0.02
Center	1 (<0.001)		1 (<0.001)	0.70	82.7	0.90
Tel Aviv	1.75 (<0.001)			0.66	74.4	0.80
Jerusalem	2.82 (0.2)	3.92 (0.007)	1 (<0.001)	0.72	85.4	0.90
South	1.55 (<0.001)	2.32 (0.01)		0.71	72.4	0.89

* EDF is the effective degree of freedom for the estimated smooth terms. Meteorological parameter units: °C for minimum temperature, mm/day for precipitation, W/m^2^ for solar radiation.

^ǂ^ Correlation coefficient between the estimated influenza-associated ILI or ARI with the observed during 2010/2011 season.

When TMIN’s median during influenza season was increased by 10% of its range (average increment of ~1.2°C), we found that influenza activity was decreased by 7.8% to 42.9% in all locations (Table 5). Increase in precipitation was followed by decreased influenza activity in Castile and León, North and Haifa (by 35.93% and 192.68%, respectively). There were no statistical changes in influenza activity in Ljubljana, Jerusalem and South. However, increase from precipitation’s 90^th^ percentile value showed a statistical increase in influenza activity in Ljubljana and South (27.7% and 15.1% respectively). An increase in SRAD by 10% of its range was associated with decreased influenza activity in Berlin (3.8%), Castile and León (16.4%), Center (17.5%), and Jerusalem (10%). There were no statistically significant changes in influenza activity in Ljubljana (Table 5).

**Table 5:** Percentage change in influenza-associated ILI or ARI per 100,000 populations when one of the meteorological variables was increased from its median value by 10% of its range during epidemic weeks (Model 2).

	Min. Temp	Precipitation	Solar Radiation
Berlin	-10.57(-15.83,-5.31)		-3.77(-6.58,-0.966)
Ljubljana	-41.71(-55.41,-28.01)	1.61(-17.36,20.58)	-3.18(-19.96,13.61)
Castile & León	-7.84(-13.04,-2.64)	-10.27(-18.71,-1.82)	-16.40(-23.22,-9.58)
North	-35.50(-41.45,-29.55)	35.93(14.98,56.89)	
Haifa	-42.85(-60.21,-25.49)	192.68(44.05,341.32)	
Center	-18.47(-25.67,-11.28)		-17.15(-23.87,-10.43)
Tel Aviv	-18.87(-24.23,-13.52)		
Jerusalem		-10.59(-25.40,4.23)	-10.00(-15.36,-4.63)
South	-22.03(-29.60,-14.47)	5.06(-15.39,25.52)	

**Fig 6:**
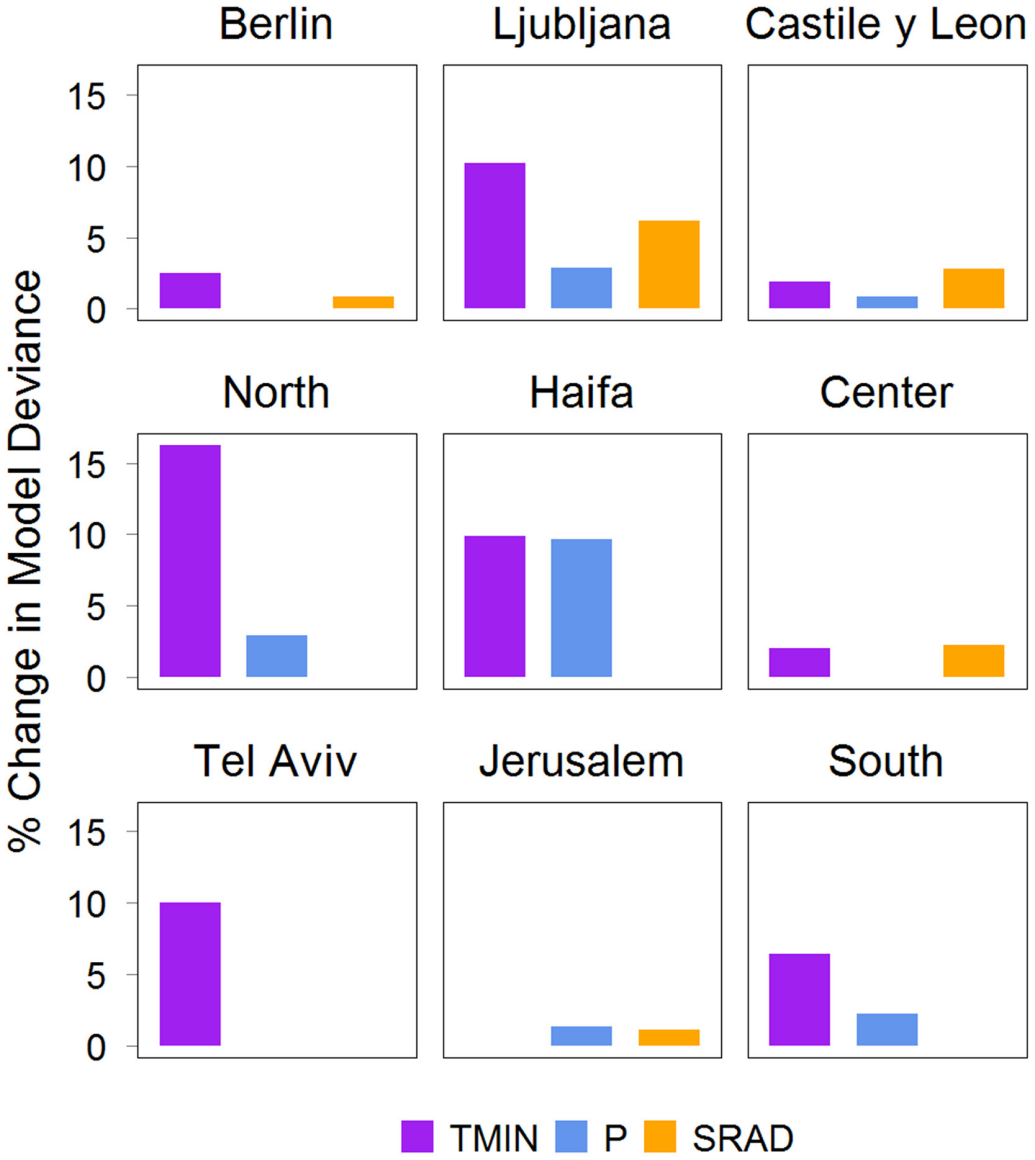
Percentage change in model deviance when the specified meteorological parameter was excluded from Model 2. TMIN is minimum temperature, PRCP is precipitation and SR is solar radiation.

When we further estimated the contribution of the meteorological covariates to the model we found that TMIN had the largest contribution (Fig 6) in Berlin, Ljubljana and Israel’s North, Haifa, Tel Aviv and South Districts (2.5%-16.2%). Similar to Model 1, SRAD had the largest contribution in Castile and León (2.8%). This pattern was also found in Jerusalem and Center Districts where SRAD contributed to 1.1% and 2.3%, respectively.

**Fig 7:**
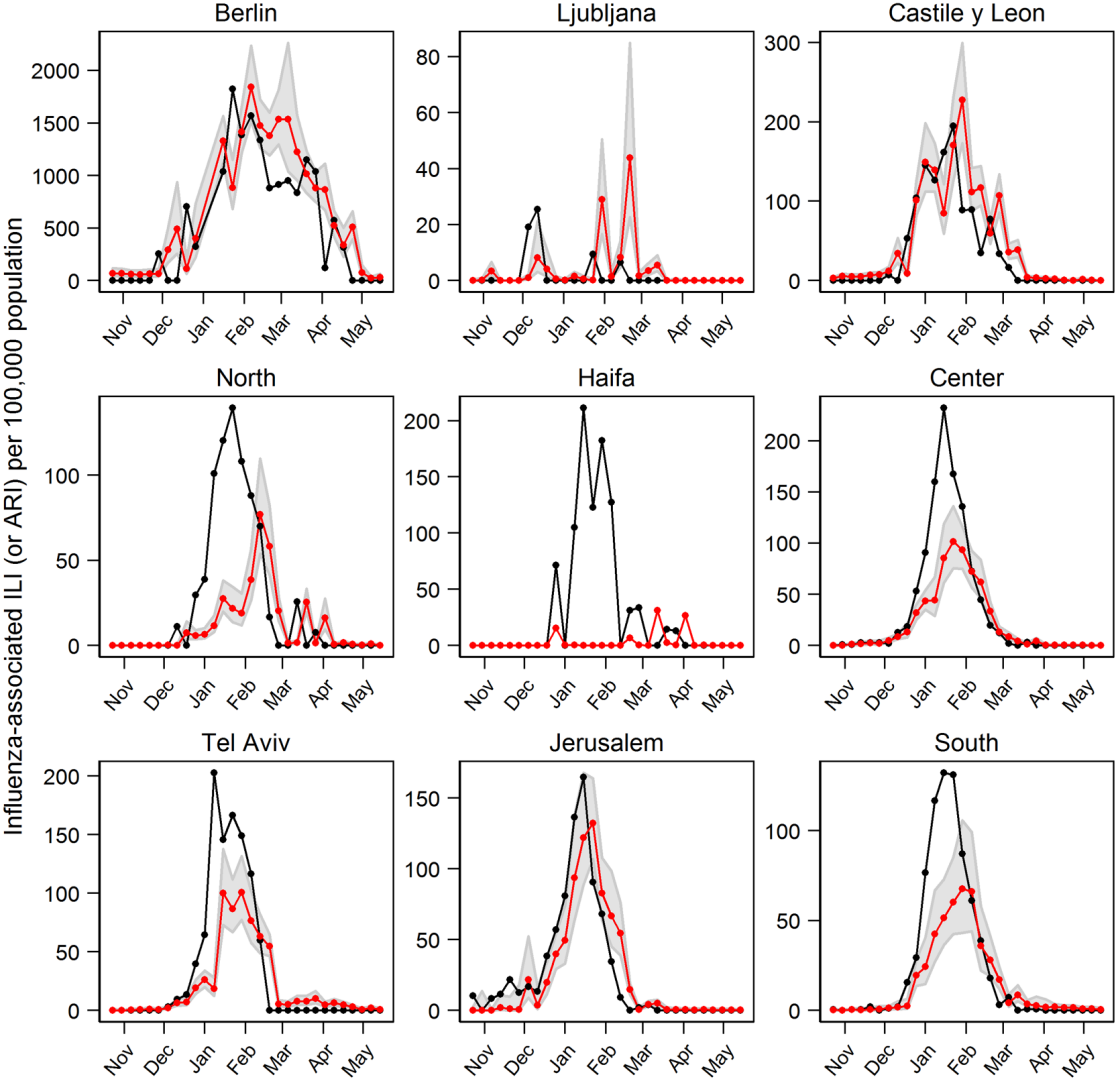
Estimated influenza activity in 2010/2011 season using Model 2 (with minimum temperature). Black line is the observations, red line is the predicted influenza and the shaded areas are the 95% CI.

TMIN models’ estimation of influenza activity in 2010/2011 could also closely follow the rise and fall of the observed influenza activity curves in more than half of the locations (Fig 7). In 3 out of the 9 locations (Castile and León, Center, and Jerusalem), the model estimated the peak week timing within 1 week of the observations (Fig 7). In Berlin and South the estimated peak week timing was within 2 weeks, while it was within 3 weeks in North and Tel Aviv. In Haifa and Ljubljana, the peak week was estimated within 8 and 10 weeks of the observed, respectively. Similar to Model 1, correlation coefficients between the observed influenza activity and the estimate from the models were lowest in Ljubljana and Haifa (less than 0.1). For other locations, the correlation coefficients ranged from 0.55 to 0.90 (Table 4). The estimated influenza activity for the training data is shown in S2 Fig.

### Model Comparison

Based on the GCV score, the TMIN model (Model 2) performed better in 3 locations (Castile and León, North and Haifa), while the SH model (Model 1) was a better model for the other 6 locations (Table 6). However, the differences in the GCV scores were relatively small (< 17% improvement). Furthermore, if we compared the model’s adjusted R-squared values, the difference between TMIN and SH models was very small (0.06 or less except in Haifa where the difference was 0.35). Both models produced very similar estimation of influenza activity during 2010/2011 season in the temperate locations (Figs 4 and 7).

**Table 6:** Difference in model performance (based on GCV score) between Model 1 and 2. Negative value indicated that the former model had better performance (lower GCV score is preferable). Model 1 is with specific humidity and Model 2 with minimum temperature.

	Δ GCV
	Model 1—Model 2
Berlin	-95.20 (-1.17%)
Ljubljana	-0.03 (-3.03%)
Castile & León	0.04 (0.83%)
North	0.07 0.57%)
Haifa	5.51 16.92%)
Center	-0.64 (-1.64%)
Tel Aviv	-7.4 (-12.36%)
Jerusalem	-0.59 (-10.94%)
South	-1.07 (-4.35%)

### Sensitivity Analysis

We tested our models using excess influenza-associated ILI or ARI, which is another frequently used influenza indicator [39–41] and defined as the amount of ILI or ARI that exceeds the baseline values (please see S1 Text for detailed descriptions and results). We found similar associations between meteorological parameters and the excess. In Model 1, SH was associated with influenza activity in all locations, whereas association with precipitation and SRAD were location-dependent (S4 Fig). Similarly, in Model 2, TMIN was associated with excess ILI (or ARI) in all locations, whereas association with precipitation and SRAD were location-dependent (S2 Table and S5 Fig).

In the main analysis, we used meteorological variables averaged over the previous 1 week (1-week lag). We further trained Model 1 and 2 using meteorological variables averaged over the previous 1 to 2 weeks and 1 to 3 weeks. Our results showed SH (in Model 1) was inversely associated (p<0.05) with influenza activity in all locations and with all average periods (S1 Appendix). TMIN (in Model 2) was inversely associated with influenza activity in all temperate locations (S2 Appendix), but not all subtropical locations. Associations with precipitation and SRAD when using all average periods remained location-dependent for both models, similar to the main results.

## Discussion

Our models (when adjusted for the previous weeks’ influenza activity) demonstrated that specific humidity (SH) was inversely associated with influenza activity in all locations while minimum temperature (TMIN) was inversely associated in all temperate locations but not all subtropical locations. Associations with precipitation and solar radiation (SRAD) were observed in the two models, but the direction of these associations was location-dependent. Taken together, our results imply that SH was an important covariate for influenza activity for these locations with temperate and subtropical climates, and TMIN was an important covariate in the temperate regions.

Consistent with our findings, epidemiological studies in the temperate regions as well as animal studies have indicated the inverse association between influenza activity and both temperature and humidity [8,10,13,24,34]. In modern societies, individuals spend most of their time indoors. Consequently, indoor humidity and temperature would primarily modulate influenza virus survivability, aerosol-borne transmission and contact transmission. Since the humidity and temperature measures used in this study were taken outdoors, the relationship revealed here may not imply the direct effect due to indoor virus survivorship or transmission efficiency. One explanation could be that the outdoor humidity and temperature may affect the indoor condition and enhance the suitability for transmission. Recent study indicated that indoor absolute humidity in school settings could be very low during winter, and that the fluctuations in the indoor humidity were primarily associated with changes in the outdoor absolute humidity [42]. Alternatively, low outdoor temperature and humidity may be sufficiently uncomfortable. This may promote indoor crowding and in turn increase the probability for contact and other modes of transmission. Finally, extreme outdoor conditions with low temperature and humidity and frequent changes in ambience from outdoor to indoor could alter the respiratory epithelium, which would facilitate the virus’ adhesion to the cell receptors [43,44].

We found that the association between influenza activity and precipitation varied across regions. The associations could be bimodal, proportional, or inversely proportional. However, the estimated contribution of precipitation to both models as measured by the percent increase in the model deviance was not only relatively low (< 9.7%), but it was also the lowest in most locations. Thus, precipitation may not be a strong covariate for influenza activity in these temperate and subtropical locations.

In both models we found that SRAD was a significant covariate in the temperate locations, but location-dependent in the subtropics. In locations where the associations was found, SRAD was inversely associated with influenza activity, consistent with another influenza study for the United States [12]. Several studies have suggested that the amount of sunshine modulates the immune system, including regulation of vitamin D, which in turn affects the susceptibility to influenza infection [18,45]. An empirical study has also suggested that sunlight’s ultraviolet radiation could increase the inactivation of influenza virus in the environment [19]. In contrast to these findings, solar radiation has very limited reach to deactivate indoor influenza virus, and a simulation study [17] demonstrated that seasonal variations in vitamin D levels were unlikely to be the principal determinant for influenza seasonality in temperate regions. Although our results indicated significant associations with SRAD in temperate and some subtropical locations, the estimated contribution to the model was not very large (< 6.2%).

The contributions of meteorological parameters to the model were relatively low—mostly less than 13%. This result was consistent with another study in European countries [20] and Netherlands [21], which used different models and covariates. Their results indicated that absolute humidity explained only 3% of the variation in influenza transmission. Despite the small contribution, absolute humidity may determine the timing for sustained transmission [21].

The resulting smooth term plots generally showed similar pattern across locations for each of the 3 meteorological parameters: specific humidity, minimum temperature and solar radiation (Figs 2 and 5). Each of them showed a downward trend, implying inverse relationship with influenza activity. There were slight variations in the plots among locations. This could be due to varying association with meteorological conditions across the temperate and the subtropics. In training the model, we have taken precautions (see Methods) to limit the flexibility of the spline functions so as to avoid overfitting that may lead to over compensation for some covariates. Moreover, the associations between these meteorological parameters and influenza activity as indicated by the smooth plots were consistent with the associations found in the literature. As there were finite samples represented by somewhat noisy time series, it was natural to find slight variations in the meteorological smooth plots. The smooth plots for precipitation noticeably varied across locations. As we previously concluded, the association with precipitation was inconclusive: its contributions to the model were lowest in most locations, and it was not a strong covariate for influenza activity.

As described in the Methods section, we excluded observations during the pandemic year as they may not well represent the typical influenza epidemics. When the models were trained with data that include observations during the pandemic year, we found different association between influenza activity and both SH and TMIN. Instead of an inverse association, we found that influenza activity increased at the lower- and higher-end values of SH and TMIN (S3 Appendix). It was previously shown [46] that in the temperate regions, higher susceptibility in a pandemic may cause an early start of the transmission in the fall when absolute humidity was not low, and that additional waves may appear in the winter when the absolute humidity became low. When pandemic year was included in our analysis, our results showed that both low and high SH and TMIN favor influenza incidence and therefore corroborated this previous finding.

We separately trained the model at each study location because our previous study in subnational locations in the tropical Central America demonstrated varying associations between influenza activity and the meteorological parameters [47]. In particular was Guatemala with subtropic-like climate that had an association more similar to the subtropic than the tropic. Because the locations in the current study span from 32°N (Tel Aviv and Jerusalem) to 52°N (Berlin), it is reasonable to assume that the influenza-meteorological association may vary across this wide latitude span. When we trained the model to all locations at the same time (S4 Appendix), we found that specific humidity, minimum temperature and solar radiation were inversely and significantly associated with influenza. However, precipitation was no longer associated with influenza activity. When the 9 subnational regions were modeled individually, precipitation showed significant association for 5 regions (SH model) and 6 regions (TMIN model). Therefore we chose to train the model at each location separately so as to understand the difference in influenza-meteorology association in this wide latitude span.

In most locations, the estimation of influenza activity during 2010/2011 season using both specific humidity and minimum temperature models closely followed the onset and the fall of the epidemic curves, though the magnitudes around the peak week were generally underestimated. Our results imply that the meteorological parameters may only be associated during the start and the end of an influenza epidemic period. Transmission propagation and its magnitude could be determined by the proportion of susceptible in the population, which was not considered in the model. The SH and TMIN models in Ljubljana, North and Haifa could not well estimate influenza activity during 2010/2011 season. The total numbers of specimens tested for influenza in these 3 locations were lower compared to other locations (Table 1), although Ljubljana had higher number of samples per population. In addition, in both Ljubljana and Haifa, there were 3 influenza peaks detected in the training period (2006 to 2010) where others had 4 peaks during this period. The low number of influenza cases in these areas may have contributed to the low model performance. Consequently, modeling accuracies for 2010/2011 influenza season in these 3 locations were not as good as others. In Ljubljana, the forecasted peak timing was off by about 2 months. In Haifa, the predicted influenza activity using TMIN model had the lowest correlation coefficient with the data (0.02), and we observed a large prediction confidence interval (not shown). This could be due to the smaller number of influenza cases and fewer peaks during the study period. Our models only include meteorological factors as covariates whereas there are other biological and socioeconomic factors, which are not easily quantified and measured, that may influence influenza activity.

We demonstrated the capability of the meteorological-based models for projecting influenza activity one week ahead using data from the previous week(s). Most of the meteorological parameters are available within hours of the observations, and meteorological forecasts are often available about 10 days before. Hence, provided that surveillance data for the previous week is available, the model could be used for operational use to make influenza activity forecast. On the other hand, although 10-day weather forecast is available, the accuracy of weather prediction decreases as prediction period increases. The accuracy of influenza forecast that depends on weather prediction is naturally affected. Hence, before 10-day weather forecast become operationally reliable, influenza prediction derived from weather prediction can serve as a reference for possible near-term influenza activity. In the event that surveillance data is not available, historical (or average from previous years) data can be used. It is possible then to forecast influenza activity 2 weeks ahead using historical average of influenza surveillance data, and a combination of meteorological observations and 10-day forecasts. Additionally, influenza models, based on historical data, such as in [48] could benefit from these meteorological covariates and, reciprocally, this model could be improved adding reliable information from the classical surveillance methods.

### Limitations

We analyzed sub-national level influenza data which evidently had smaller populations and influenza samples. This was a factor that may affect the statistical significance of the results. However, the meteorological condition within a subnational location often had less variability, which provided a better setting to understand the meteorological association with influenza. Since our subnational data was limited to 9 study locations, this may hinder generalizing our result to other similar locations. Nonetheless, despite these limitations, consistency of our results with others in literature demonstrates the robustness of our approach and findings.

The influenza indicator used in this study assumed [49] that samples from the virological surveillance were representative of the ILI or ARI patients in the clinical surveillance. This assumption may not be entirely accurate in reality. Differences in the surveillance method across the 4 countries studied (S1 Text) may also affect how close the indicator in representing influenza activity. We have also conducted the analysis using another commonly-used influenza indicator, excess ILI or ARI, which showed consistent results with our main finding. We did not use the latter indicator because the epidemic week definition that was needed in the calculation varied across countries. Moreover, an accurate estimation of such indicator typically required more than 5 years of data [48] and the computation was sensitive to the periods of observations included [50]. Despite its limitations, the current influenza indicator was the most suitable for all study locations.

There are a multitude of factors that contribute to influenza transmission—from behavioral, susceptibility, socioeconomic to meteorological conditions. In this study, we only considered meteorological conditions and previous weeks’ influenza activity as covariates. Susceptibility was not a covariate per se, but was reflected in previous week’s influenza activity, which was one of the covariates. The susceptible population was also implicitly considered as the influenza activity was constrained by the total, the recovered, and the susceptible populations. Other factors contributing to influenza transmission were not easily measured and quantified. These unaccounted factors may contribute to the differences between the predicted and observed influenza activity as seen in Figs 4 and 7.

Although the models only considered a subset of the factors that affect influenza transmission, the onset and the fall of the epidemic curves were still accurately estimated, and the timing of the peak was closely estimated for most locations. The association between influenza activity and meteorological parameters inferred by the models were also consistent with finding in literature that used different modeling techniques.

Our findings only showed associations between influenza activity and meteorological parameters and does not necessarily imply a causal relationship. However, these findings may suggest the meteorological parameters that warrant further testing for causal relationship. Another limitation to this study was the use of outdoor measurements where most people in modern societies spend much of their time indoors. Lastly, other respiratory viruses may co-circulate with influenza virus—such as Respiratory Syncytial Virus (RSV), adenovirus, parainfluenza virus and so on—which could vary with meteorological parameters. Since there was no sufficient data to include the co-circulating respiratory viruses in the models, this is a potential limitation of our study.

## Conclusion

Our study demonstrated significant association between specific humidity and influenza activity across the 9 locations with temperate or subtropical climates. Such association at the subnational level had frequently been found in the continental United States [10,34], but further studies in the other temperate and subtropical regions are needed to extend the validity of the association. Our study also indicated that the meteorological-based estimation of influenza activity in most locations showed a good agreement with the observed data, especially during the start and the end of an epidemic period. Therefore integrating meteorological parameters for influenza forecasting in the surveillance system may benefit the public health efforts in reducing the burden of seasonal influenza. More studies are necessary to understand the role of these parameters in the viral transmission and host susceptibility process.

## Supporting Information

S1 Appendix Specific Humidity Model with Different Meteorological Parameters Average Periods. (PDF)

S2 Appendix Minimum Temperature Model with Different Meteorological Parameters Average Periods. (PDF)

S3 Appendix Models with Observations from Pandemic Year Included in Training Dataset. (PDF)

S4 Appendix Models Trained to All Locations Simultaneously. (PDF)

S1 Fig Predicted influenza activity for training data using Model 1 (with Specific Humdity). Black line is the observation and red line is the model estimate.
(TIF)

S2 Fig Predicted influenza activity for training data using Model 2 (with minimum temperature). Black line is the observation and red line is the model estimate.
(TIF)

S3 Fig Estimated ILI or ARI baseline for each study location. (TIF)

S4 Fig Meteorological smooth terms for specific humidity model (Model 1) with excess ILI or ARI as dependent variable. The y-axis is log(Y) and normalized. x-axis is the value of the meteorological variable.
(TIF)

S5 Fig Estimated excess ILI or ARI in 2010/2011 season using specific humidity model (Model 1). Black line is the observations, red line is the predicted influenza, grey lines are the 95% CI.
(TIF)

S6 Fig Meteorological smooth terms for minimum temperature model (Model 2) with excess ILI or ARI as dependent variable. The y-axis is log(Y) and normalized. x-axis is the value of the meteorological variable.
(TIF)

S7 Fig Estimated excess ILI or ARI in 2010/2011 season using specific humidity model (Model 1). Black line is the observations, red line is the predicted influenza, and grey lines are the 95% CI.
(TIF)

S1 Table Regression model for excess ILI or ARI (Model 1 with specific humidity) (DOCX)

S2 Table Regression model for excess ILI or ARI (Model 2 with minimum temperature) (DOCX)

S1 Text Details on analytic approach, meteorological data and supplementary results. (DOCX)
