# Supplementary material for: Associations between Meteorological Parameters and Influenza Activity in Berlin (Germany), Ljubljana (Slovenia), Castile and León (Spain) and Israeli Districts
Source: PLoS One. 2015 Aug 26;10(8):e0134701. doi: 10.1371/journal.pone.0134701 (PMC4550247; doi:10.1371/journal.pone.0134701)
Supplement: S1 Appendix — (PDF) [file pone.0134701.s001.pdf]

S1 APPENDIX

Specific Humidity Model with Different Meteorological Parameters Average Periods

## 1 Meteorological variables averaged from previous 1 to 3 weeks

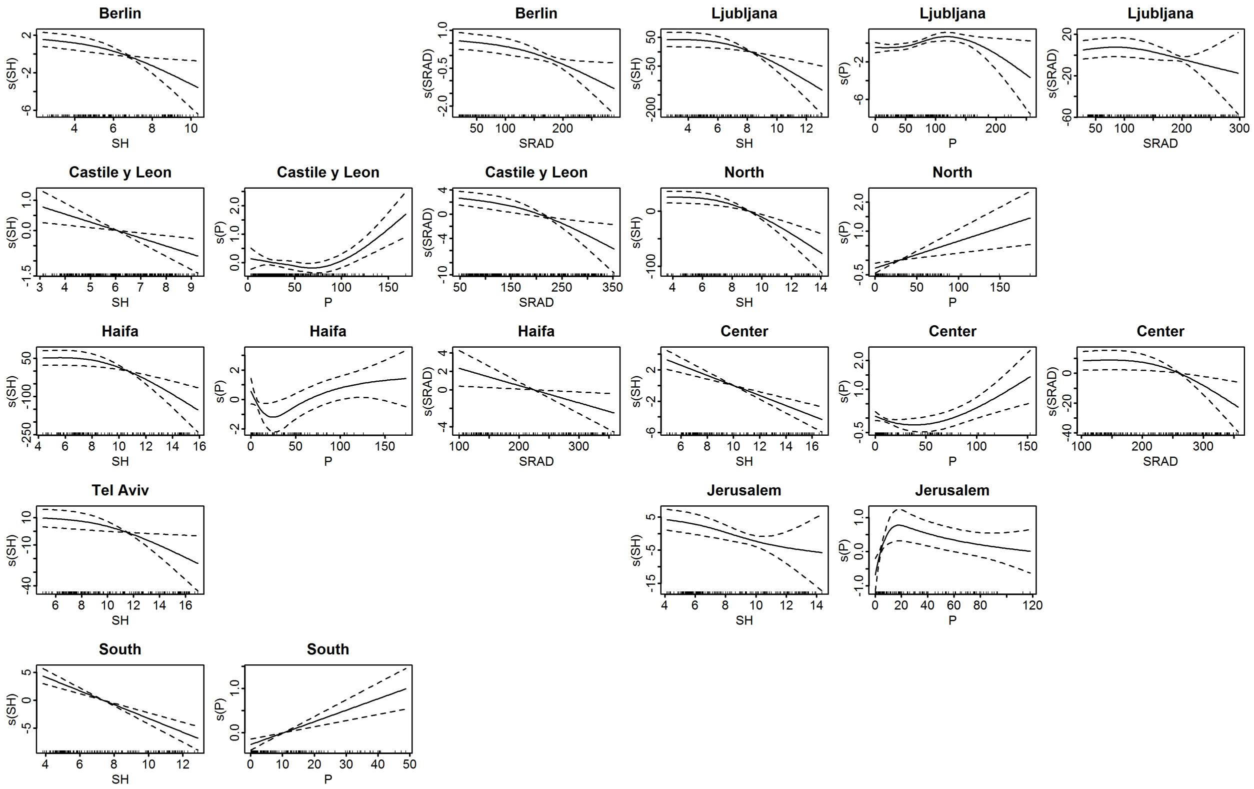

Fig. A. Resulting meteorological smooth terms. Only terms that are significant are plotted. The y-axis is in the log scale and normalized, x-axis is the value of the meteorological variable. SH = specific humidity, P = Precipitation, SRAD = Solar Radiation.

Table A. Meteorological smooth term effective degree of freedom and p-value in parenthesis

| | Specific Humidity | Precipitation | Solar Radiation | Adj.Rsq | % Dev. Expl. |
|---|---|---|---|---|---|
| Berlin | 1.69 (3e-05) | | 1.56 (0.02) | 0.73 | 77.6 |
| Ljubljana | 1.89 (3e-05) | 2.77 (0.02) | 1.96 (0.02) | 0.41 | 67.8 |
| Castile Y Leon | 1 (0.003) | 2.38 (0.006) | 1.75 (2e-09) | 0.56 | 72.5 |
| North | 1.92 (4e-11) | 1 (0.002) | | 0.45 | 72.2 |
| Haifa | 1.92 (6e-04) | 2.82 (2e-04) | 1 (0.02) | 0.33 | 64.1 |
| Center | 1 (2e-07) | 1.83 (0.006) | 1.89 (0.03) | 0.62 | 85.1 |
| Tel Aviv | 1.76 (4e-09) | | | 0.61 | 77.5 |
| Jerusalem | 1.83 (2e-05) | 2.89 (7e-05) | | 0.77 | 88.5 |
| South | 1 (1e-09) | 1 (3e-05) | | 0.55 | 73.8 |

## 2 Meteorological variables averaged from previous 1 to 2 weeks

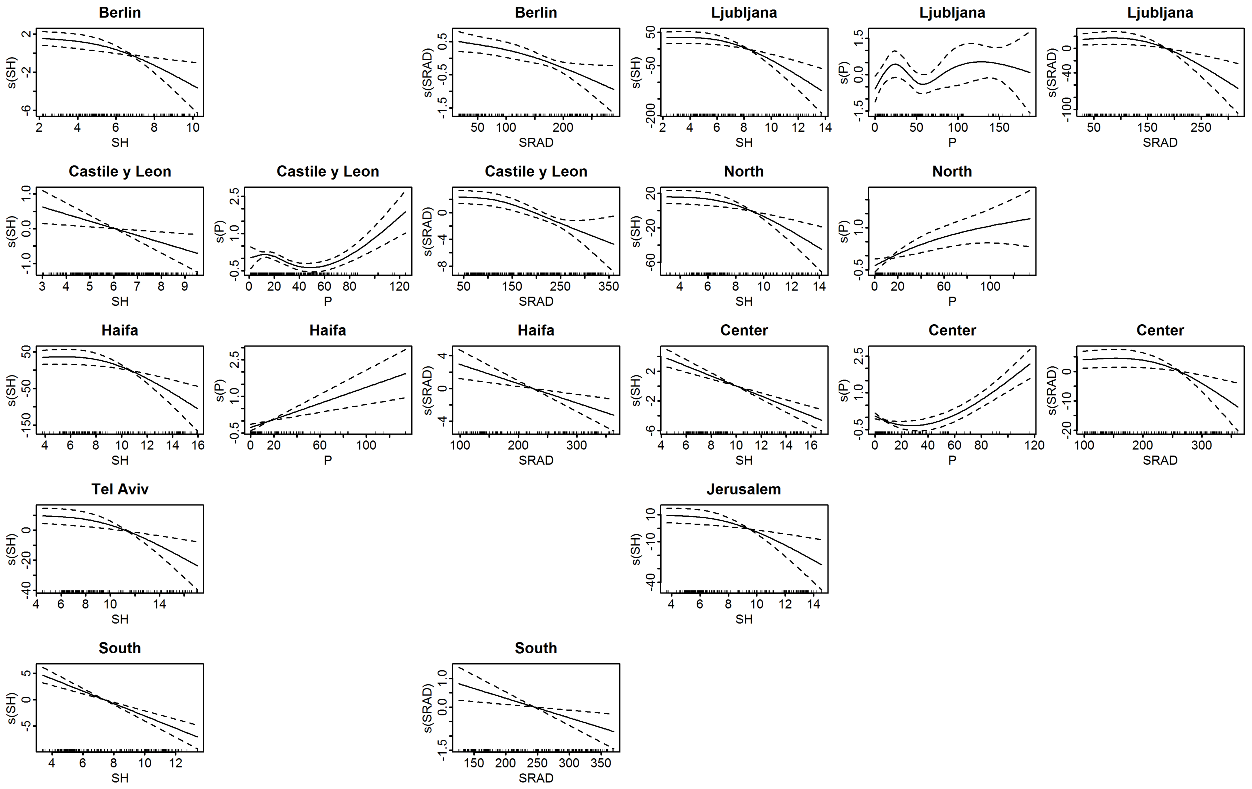

Fig. B. Resulting meteorological smooth terms. Only terms that are significant are plotted. The y-axis is in the log scale and normalized, x-axis is the value of the meteorological variable. SH = specific humidity, P = Precipitation, SRAD = Solar Radiation.

Table B. Meteorological smooth term effective degree of freedom and p-value in parenthesis

| | Specific Humidity | Precipitation | Solar Radiation | Adj.Rsq | % Dev. Expl. |
|---|---|---|---|---|---|
| Berlin | 1.69 (3e-05) | | 1.56 (0.02) | 0.73 | 77.6 |
| Ljubljana | 1.92 (4e-04) | 3.77 (0.03) | 1.91 (0.007) | 0.37 | 67.9 |
| Castile Y Leon | 1 (0.009) | 2.69 (4e-05) | 1.99 (1e-08) | 0.57 | 72.9 |
| North | 1.87 (6e-11) | 1.43 (0.007) | | 0.45 | 72.2 |
| Haifa | 1.91 (2e-04) | 1 (1e-04) | 1 (0.001) | 0.36 | 60.7 |
| Center | 1 (2e-09) | 1.96 (6e-11) | 1.9 (0.01) | 0.87 | 88.0 |
| Tel Aviv | 1.81 (2e-09) | | | 0.64 | 77.6 |
| Jerusalem | 1.84 (8e-09) | | | 0.73 | 86.6 |
| South | 1 (2e-09) | | 1 (0.005) | 0.57 | 74.0 |
