# Supplementary material for: Associations between Meteorological Parameters and Influenza Activity in Berlin (Germany), Ljubljana (Slovenia), Castile and León (Spain) and Israeli Districts
Source: PLoS One. 2015 Aug 26;10(8):e0134701. doi: 10.1371/journal.pone.0134701 (PMC4550247; doi:10.1371/journal.pone.0134701)
Supplement: S2 Appendix — (PDF) [file pone.0134701.s002.pdf]

# S2 APPENDIX
Minimum Temperature Model with Different Meteorological Parameters Average Periods

## 1 Meteorological variables averaged from previous 1 to 3 weeks

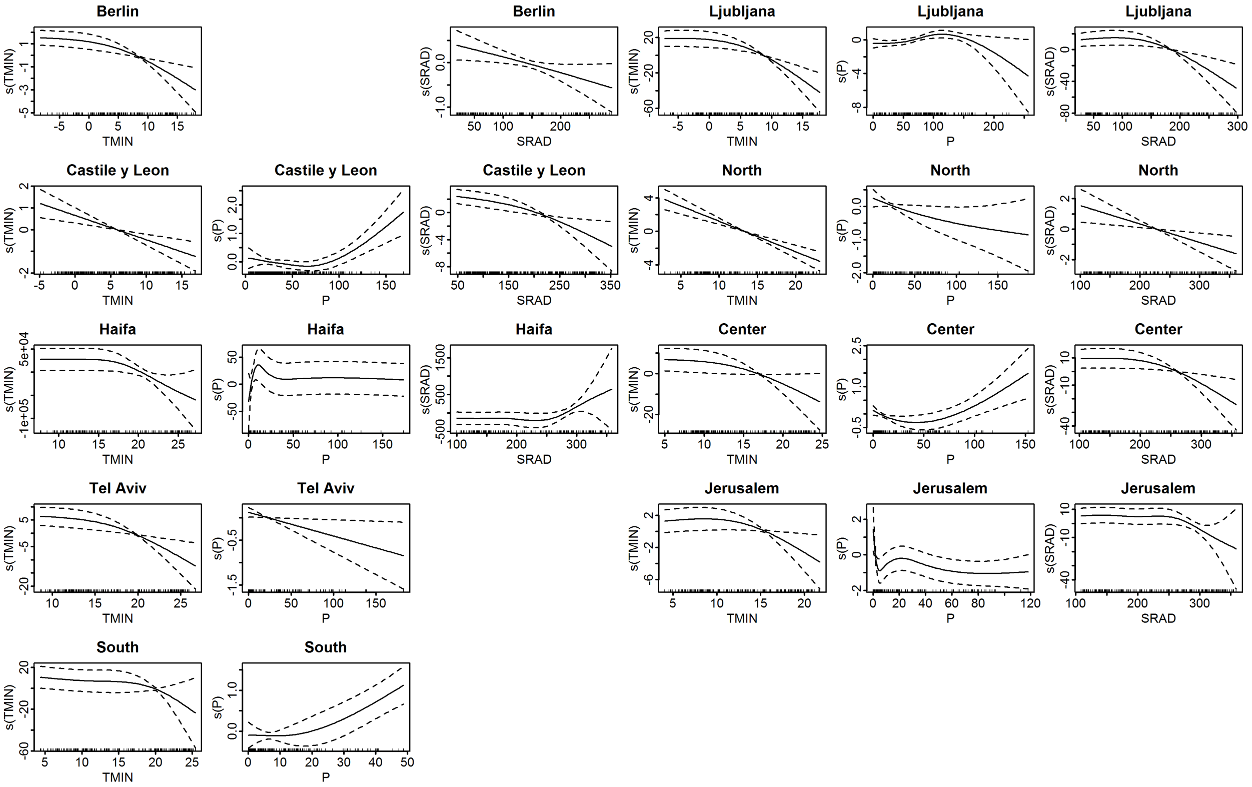


Fig. A. Resulting meteorological smooth terms. Only terms that are significant are plotted. The y-axis is in the log scale and normalized, x-axis is the value of the meteorological variable. TMIN = minimum temperature, P = Precipitation, SRAD = Solar Radiation.

Table A. Meteorological smooth term effective degree of freedom and p-value in parenthesis

| | Minimum Temperature | Precipitation | Solar Radiation | Adj.Rsq | % Dev. Expl. |
|---|---|---|---|---|---|
| Berlin | 1.8 (1e-04) | | 1.05 (0.04) | 0.72 | 77.2 |
| Ljubljana | 1.93 (6e-07) | 2.77 (0.04) | 1.91 (9e-04) | 0.44 | 68.8 |
| Castile Y Leon | 1 (3e-04) | 2.3 (0.01) | 1.69 (2e-08) | 0.56 | 72.9 |
| North | 1 (3e-09) | 1.23 (0.05) | 1 (0.005) | 0.45 | 70.1 |
| Haifa | 2.64 (9e-06) | 3.98 (4e-05) | 3.86 (4e-05) | 0.52 | 83.4 |
| Center | 1.7 (0.002) | 1.84 (0.002) | 1.88 (0.03) | 0.64 | 84.6 |
| Tel Aviv | 1.82 (2e-06) | 1 (0.02) | | 0.58 | 75.8 |
| Jerusalem | 1.85 (0.05) | 3.91 (3e-05) | 3.85 (4e-04) | 0.80 | 88.6 |
| South | 2.75 (8e-13) | 1.68 (2e-05) | 2.02 (0.1) | 0.72 | 78.3 |

## 2 Meteorological variables averaged from previous 1 to 2 weeks

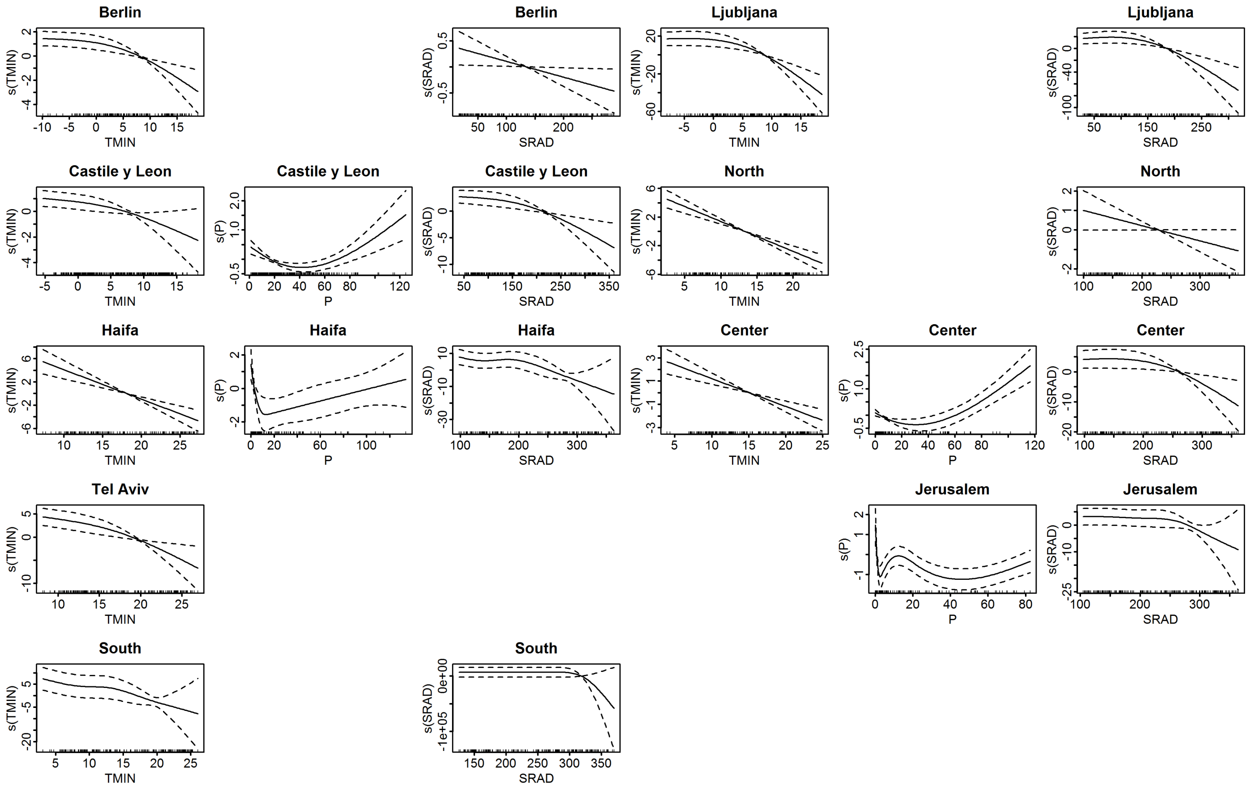

Fig. B. Resulting meteorological smooth terms. Only terms that are significant are plotted. The y-axis is in the log scale and normalized, x-axis is the value of the meteorological variable. TMIN = minimum temperature, P = Precipitation, SRAD = Solar Radiation.

Table B. Meteorological smooth term effective degree of freedom and p-value in parenthesis

| | Minimum Temperature | Precipitation | Solar Radiation | Adj.Rsq | % Dev. Expl. |
|---|---|---|---|---|---|
| Berlin | 1.81 (1e-04) | | 1 (0.03) | 0.72 | 77.2 |
| Ljubljana | 1.95 (5e-08) | | 1.94 (0.001) | 0.41 | 70.9 |
| Castile Y Leon | 1.49 (0.002) | 1.94 (1e-04) | 1.81 (4e-07) | 0.56 | 72.7 |
| North | 1 (1e-11) | 1 (0.08) | 1 (0.05) | 0.56 | 73.1 |
| Haifa | 1 (8e-07) | 2.88 (0.004) | 2.99 (0.002) | 0.52 | 65.8 |
| Center | 1 (1e-06) | 1.95 (2e-08) | 1.88 (0.03) | 0.87 | 87.0 |
| Tel Aviv | 1.73 (1e-07) | 1.74 (0.1) | | 0.67 | 76.1 |
| Jerusalem | 1.81 (0.06) | 3.98 (1e-08) | 3.67 (0.004) | 0.79 | 88.1 |
| South | 2.89 (0.004) | | 4.78 (7e-11) | 0.76 | 80.4 |
