# Supplementary material for: Associations between Meteorological Parameters and Influenza Activity in Berlin (Germany), Ljubljana (Slovenia), Castile and León (Spain) and Israeli Districts
Source: PLoS One. 2015 Aug 26;10(8):e0134701. doi: 10.1371/journal.pone.0134701 (PMC4550247; doi:10.1371/journal.pone.0134701)
Supplement: S3 Appendix — (PDF) [file pone.0134701.s003.pdf]

S3 APPENDIX

Models with Observations from Pandemic Year Included in Training Dataset

A. Specific humidity model

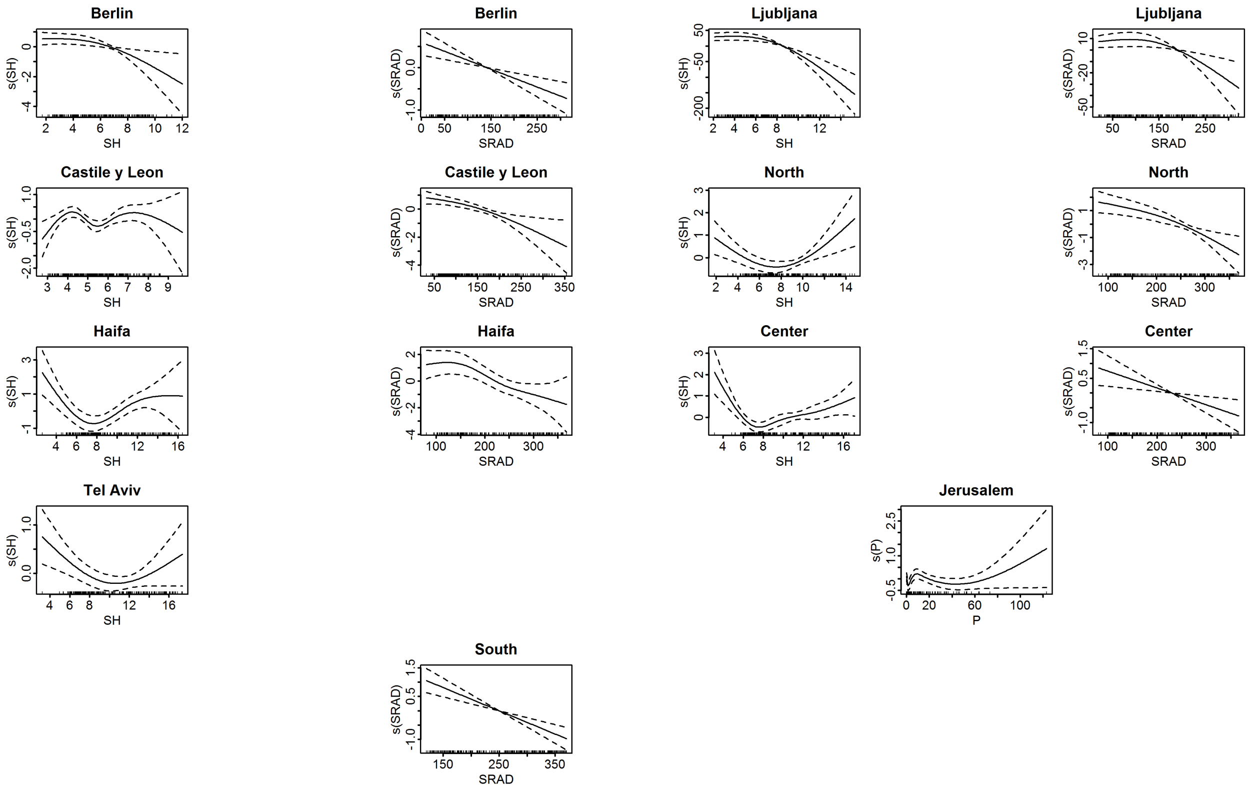

Fig. A. Resulting meteorological smooth terms. Only terms that are significant are plotted. The y-axis is in the log scale and normalized, x-axis is the value of the meteorological variable. SH = Specific Humidity, P = Precipitation, SRAD = Solar Radiation.

Table A. Meteorological smooth term effective degree of freedom and p-value in parenthesis

	Specific Humidity	Precipitation	Solar Radiation
Berlin	1.73 (0.006)		1 (1e-04)
Ljubljana	1.99 (1e-05)	1.69 (0.06)	1.92 (0.007)
Castile Y Leon	3.74 (0.04)		1.53 (5e-05)
North	1.97 (0.002)		1.67 (0.001)
Haifa	2.79 (2e-04)	1 (0.06)	2.64 (0.02)
Center	3.62 (4e-06)	1 (0.08)	1 (0.005)
Tel Aviv	1.83 (0.01)		1.05 (0.05)
Jerusalem	1.81 (0.6)	3.85 (0.008)	1.44 (0.1)
South			1 (1e-06)

B. Minimum temperature model

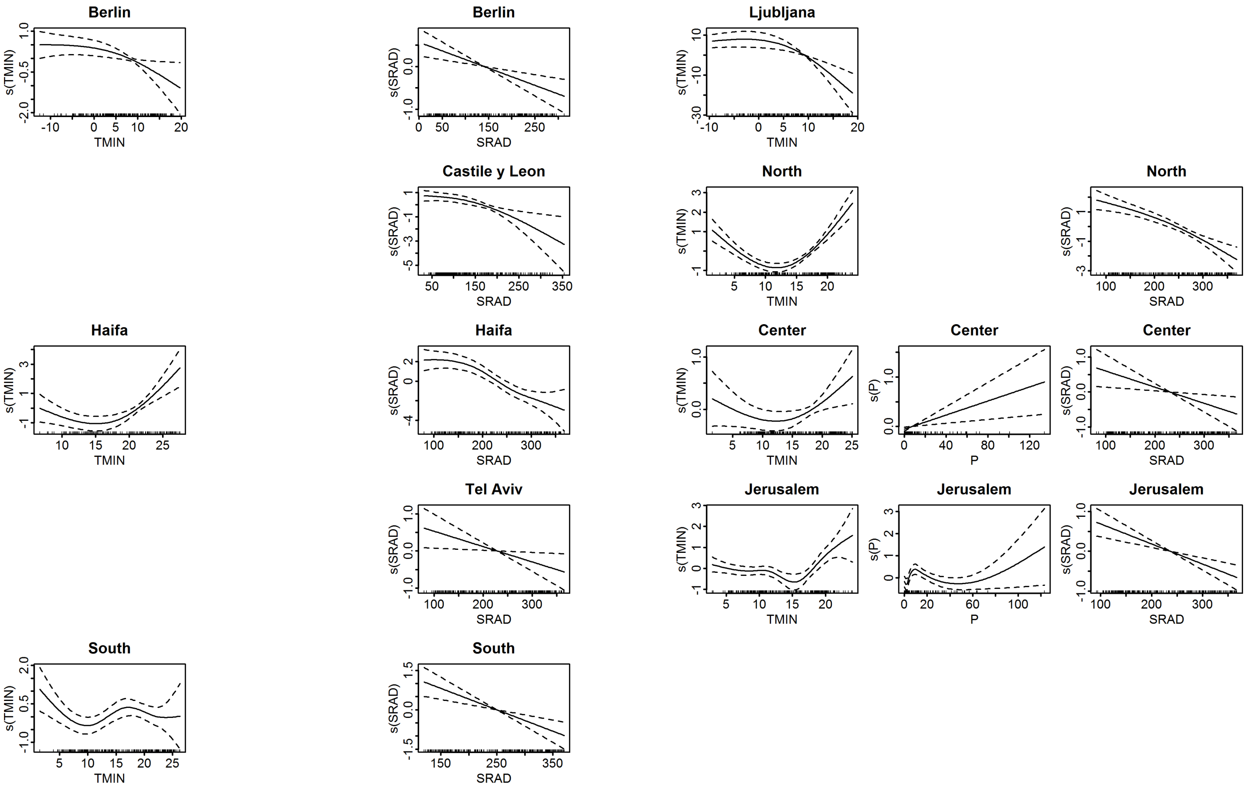

Fig. B. Resulting meteorological smooth terms. Only terms that are significant are plotted. The y-axis is in the log scale and normalized, x-axis is the value of the meteorological variable. TMIN = minimum temperature, P = Precipitation, SRAD = Solar Radiation.

Table B. Meteorological smooth term effective degree of freedom and p-value in parenthesis

	Minimum Temperature	Precipitation	Solar Radiation
Berlin	1.65 (0.04)		1 (5e-04)
Ljubljana	1.93 (2e-04)		1.84 (0.05)
Castile Y Leon			1.69 (1e-04)
North	1.99 (3e-13)		1.65 (8e-08)
Haifa	1.97 (2e-04)		2.88 (6e-06)
Center	1.76 (0.03)	1 (0.007)	1 (0.01)
Tel Aviv	2.74 (0.5)		1 (0.02)
Jerusalem	4.48 (4e-04)	4.98 (1e-04)	1 (4e-05)
South	3.2 (0.003)		1 (2e-04)
