# Supplementary material for: Associations between Meteorological Parameters and Influenza Activity in Berlin (Germany), Ljubljana (Slovenia), Castile and León (Spain) and Israeli Districts
Source: PLoS One. 2015 Aug 26;10(8):e0134701. doi: 10.1371/journal.pone.0134701 (PMC4550247; doi:10.1371/journal.pone.0134701)
Supplement: S4 Appendix — (PDF) [file pone.0134701.s004.pdf]

S4 APPENDIX
Models Trained to All Locations Simultaneously

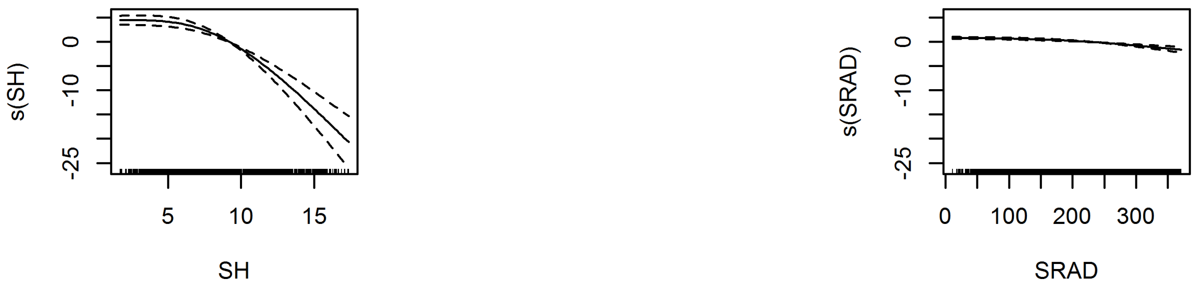

Fig. A. Resulting meteorological smooth terms for Specific Humidity model. The y-axis is in the log scale and normalized, x-axis is the value of the meteorological variable. SH = Specific Humidity, P = Precipitation, SRAD = Solar Radiation.

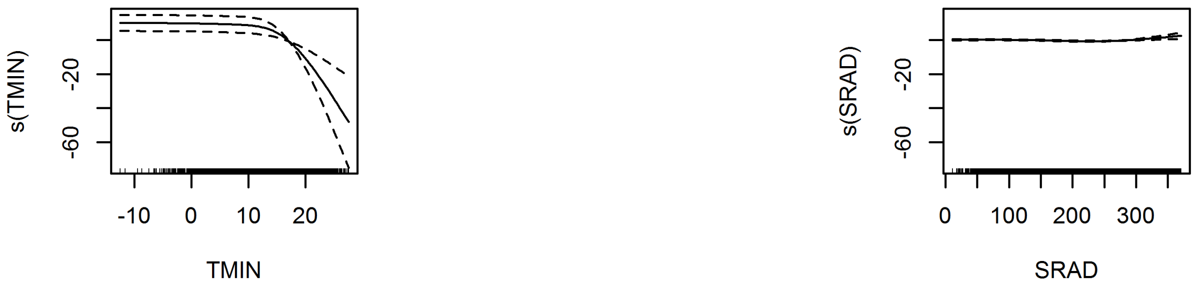

Fig. B. Resulting meteorological smooth terms for Minimum Temperature model. The y-axis is in the log scale and normalized, x-axis is the value of the meteorological variable. TMIN = Minimum Temperature, P = Precipitation, SRAD = Solar Radiation.

Table A. Meteorological smooth term effective degree of freedom and p-value in parenthesis. SH = Specific Humidity, TMIN = Minimum Temperature, P = Precipitation, SRAD = Solar Radiation

	SH	TMIN	P	SRAD	Adj.Rsq	% Dev. Expl.
SH Model	1.97 (<2e-16)			1.9 (9e-16)	0.78	78.0
TMIN Model		2.91 (<2e-16)		2.91 (1e-09)	0.79	78.1
