# Supplementary figures and images for: Associations between Meteorological Parameters and Influenza Activity in Berlin (Germany), Ljubljana (Slovenia), Castile and León (Spain) and Israeli Districts
Source: PLoS One. 2015 Aug 26;10(8):e0134701. doi: 10.1371/journal.pone.0134701 (PMC4550247; doi:10.1371/journal.pone.0134701)

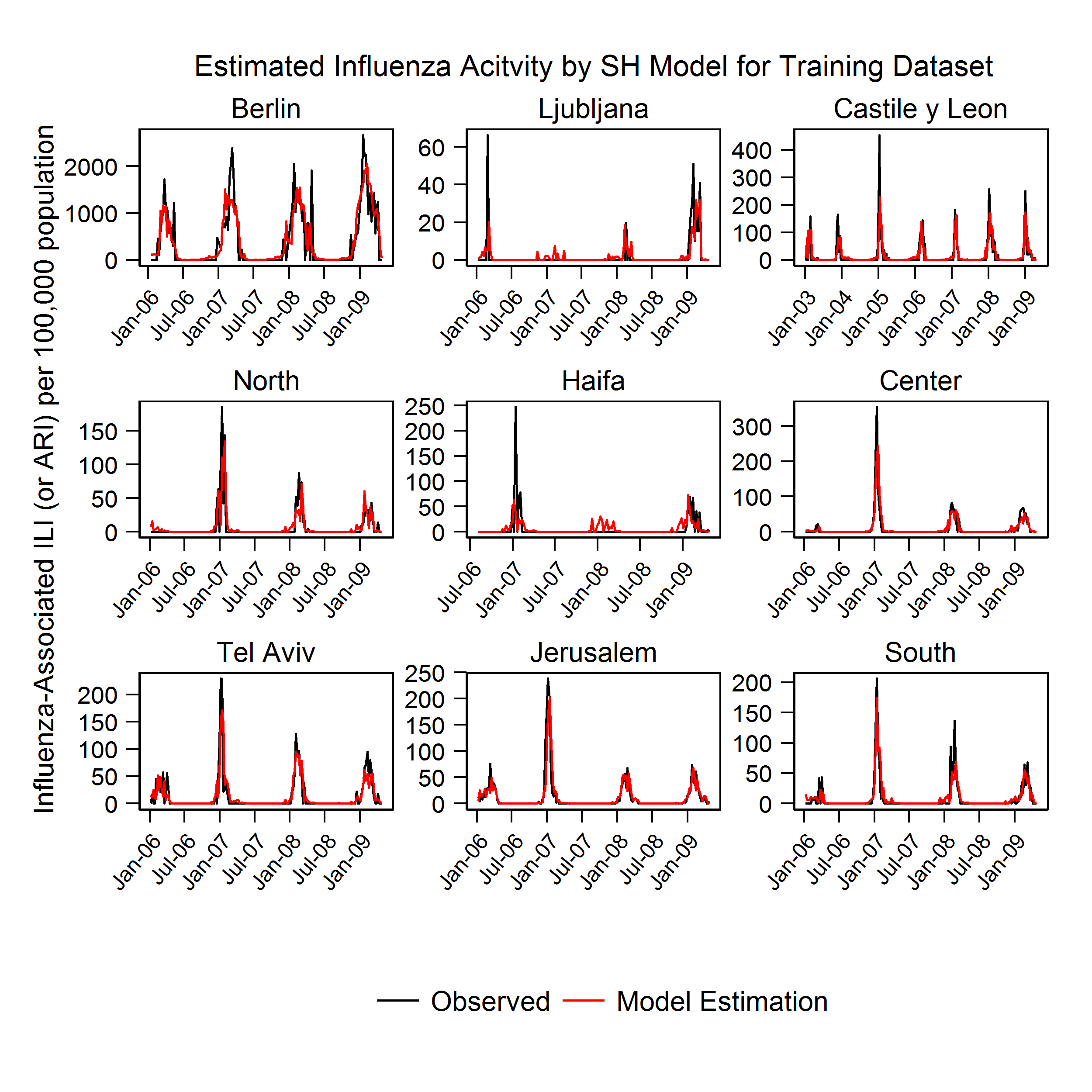

Supplement: S1 Fig — Black line is the observation and red line is the model estimate. (TIF) [file pone.0134701.s005.tif]

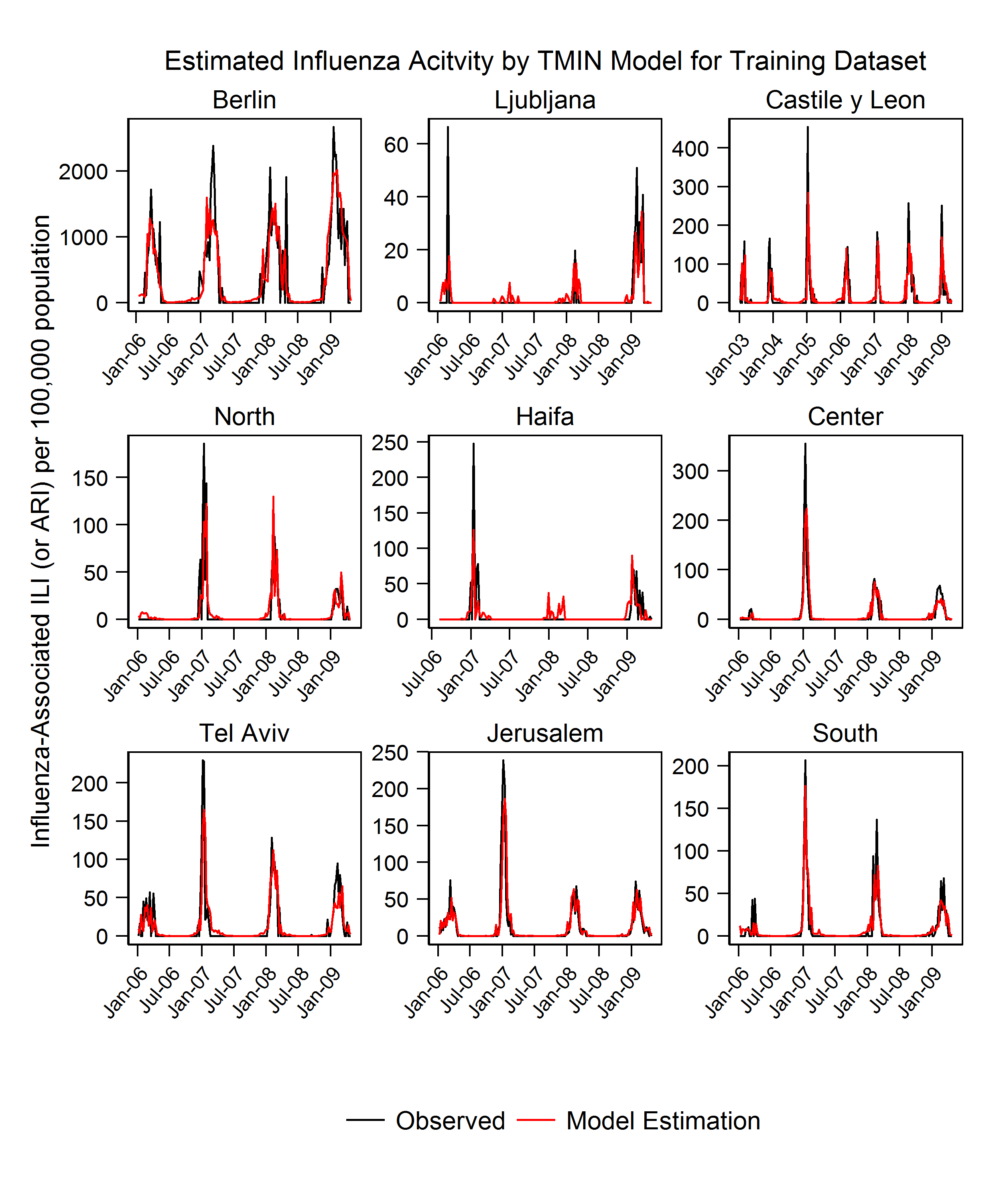

Supplement: S2 Fig — Black line is the observation and red line is the model estimate. (TIF) [file pone.0134701.s006.tif]

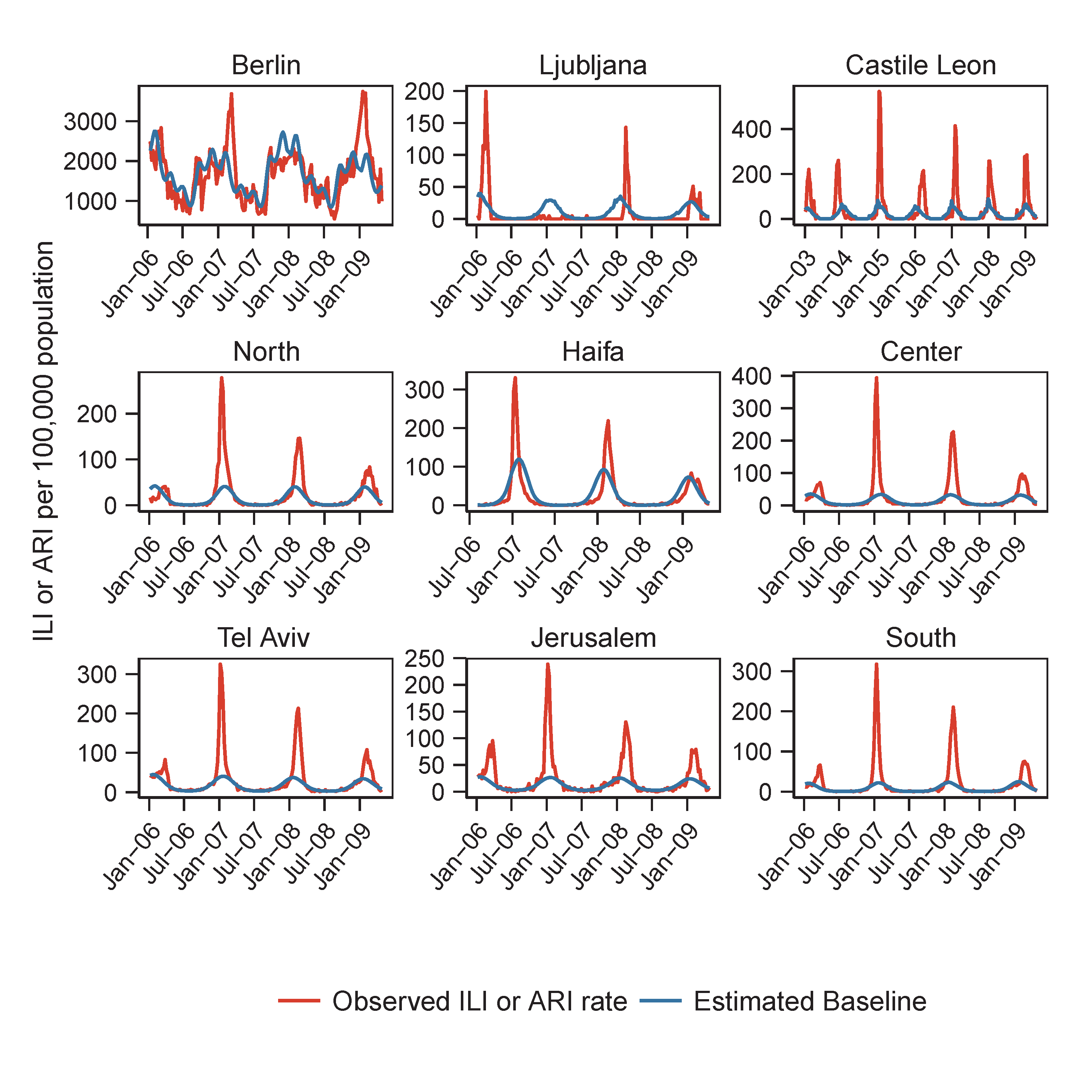

Supplement: S3 Fig — (TIF) [file pone.0134701.s007.tif]

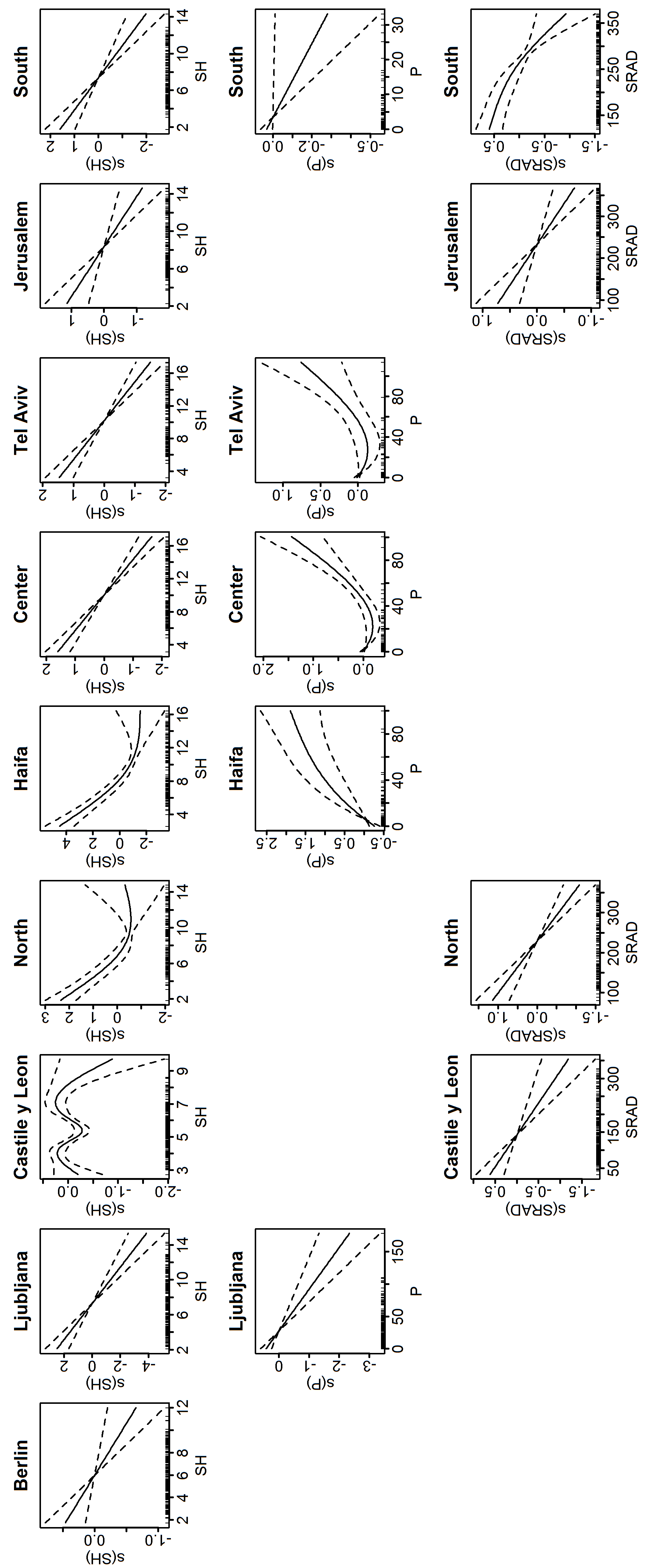

Supplement: S4 Fig — The y-axis is log(Y) and normalized. x-axis is the value of the meteorological variable. (TIF) [file pone.0134701.s008.tif]

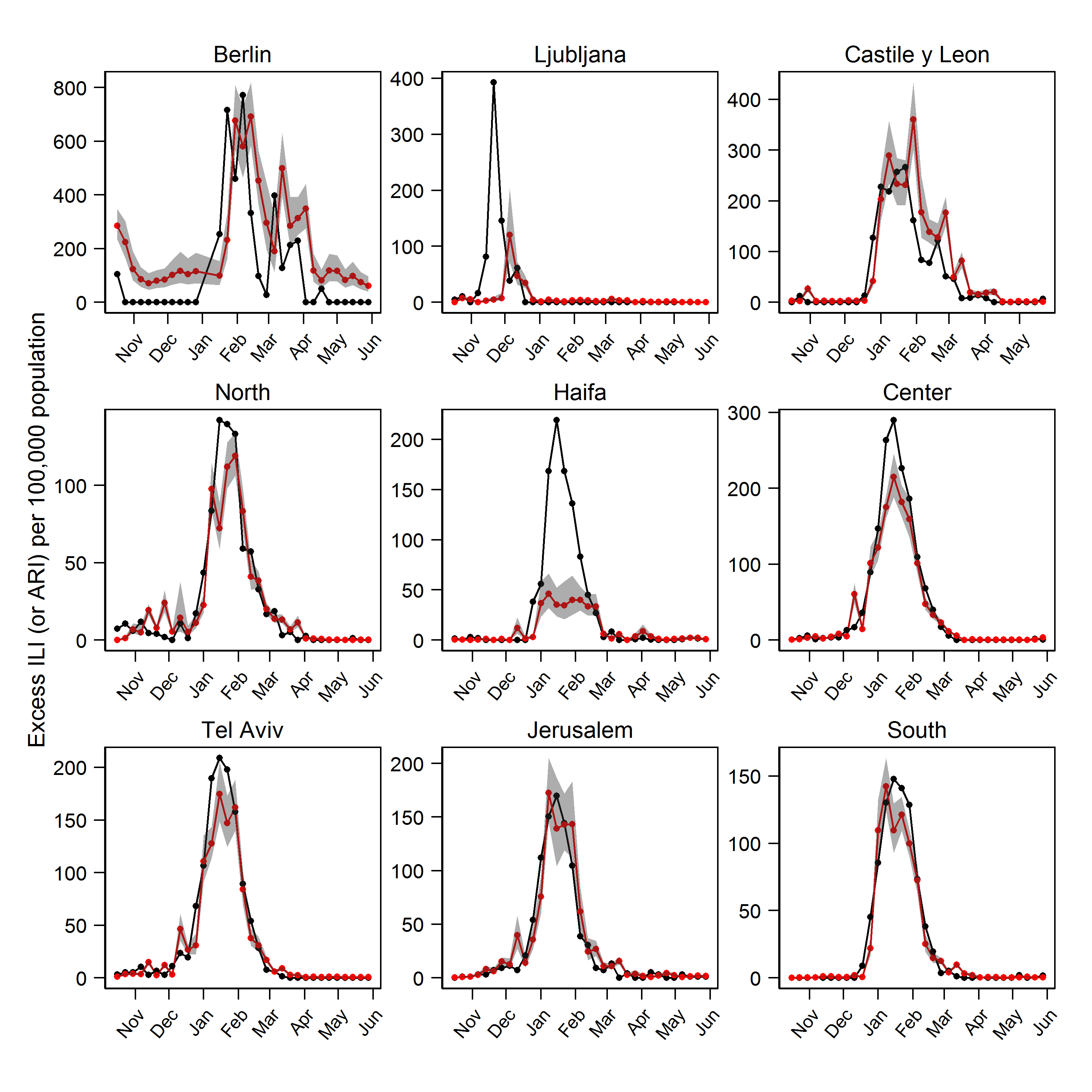

Supplement: S5 Fig — Black line is the observations, red line is the predicted influenza, grey lines are the 95% CI. (TIF) [file pone.0134701.s009.tif]

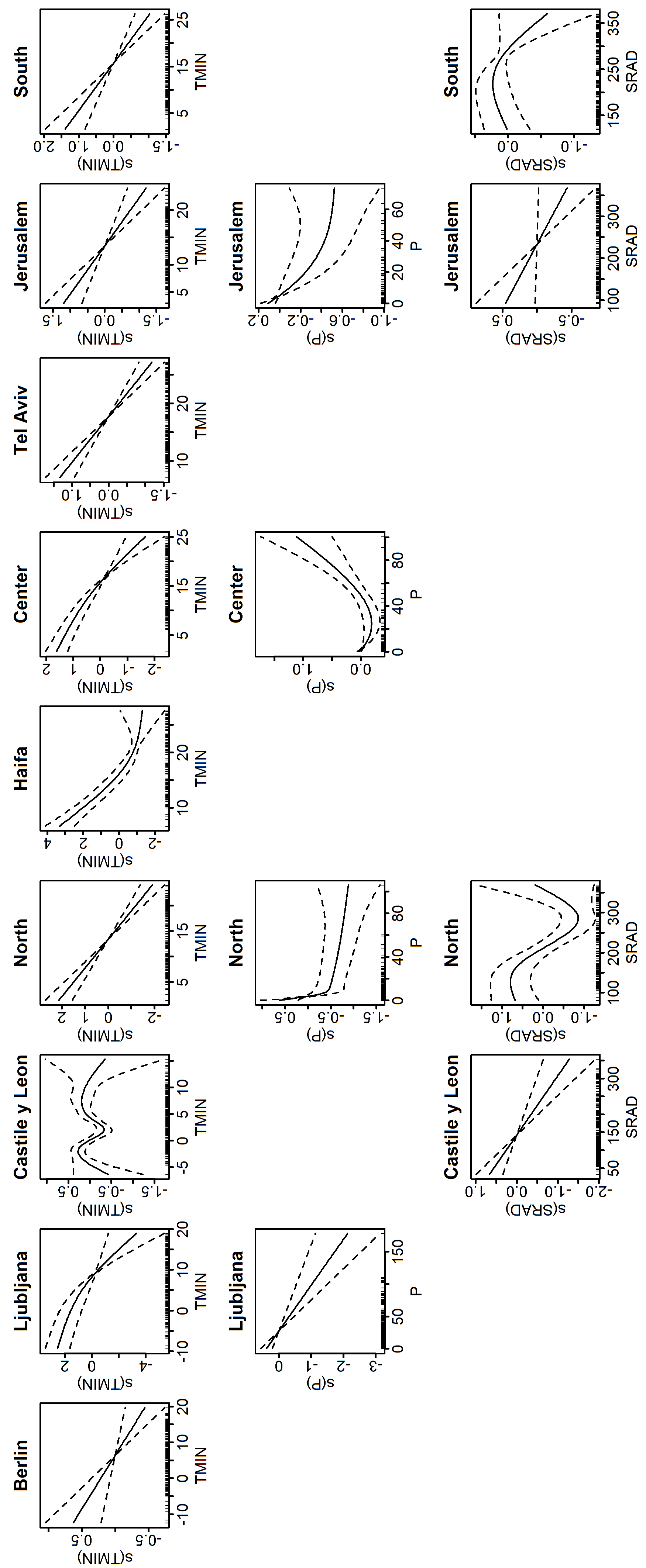

Supplement: S6 Fig — The y-axis is log(Y) and normalized. x-axis is the value of the meteorological variable. (TIF) [file pone.0134701.s010.tif]

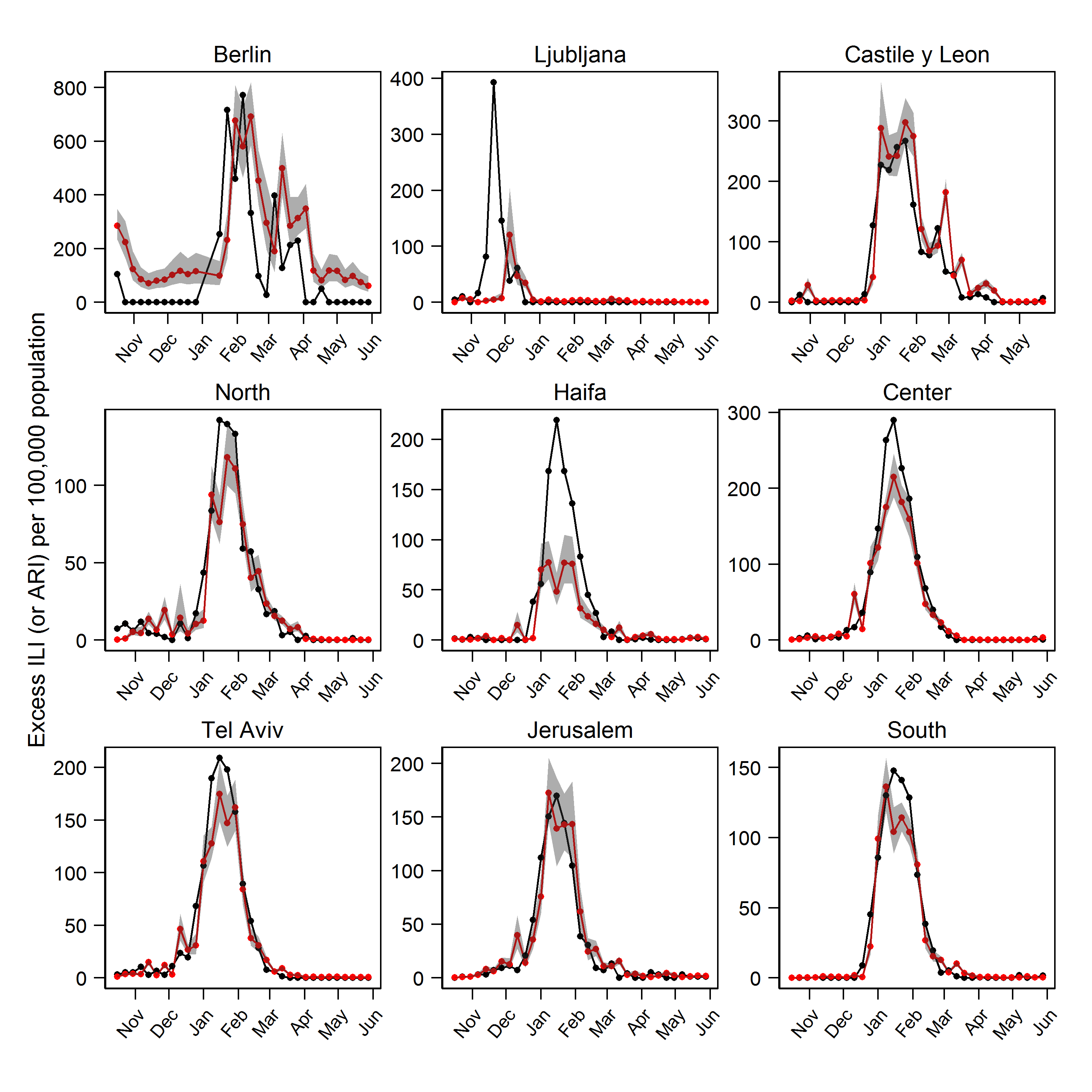

Supplement: S7 Fig — Black line is the observations, red line is the predicted influenza, and grey lines are the 95% CI. (TIF) [file pone.0134701.s011.tif]
