# Supplementary material for: Associations between Meteorological Parameters and Influenza Activity in Berlin (Germany), Ljubljana (Slovenia), Castile and León (Spain) and Israeli Districts
Source: PLoS One. 2015 Aug 26;10(8):e0134701. doi: 10.1371/journal.pone.0134701 (PMC4550247; doi:10.1371/journal.pone.0134701)
Supplement: S1 Table — (DOCX) [file pone.0134701.s012.docx]

S1 Table. Regression model for excess ILI or ARI (Model 1 with specific humidity)

| **Location** | **Meteorological Smooth Terms EDF (p-value)*** | | | **Adj. R^2^** | **% Dev. Explained** | **Pred. Corr. Coeff.**^ǂ^ |
| --- | --- | --- | --- | --- | --- | --- |
|  | **Specific Humidity** | **Precipitation** | **Solar Radiation** |  |  |  |
| Berlin | 1 (0.004) |  |  | 0.49 | 42 | 0.56 |
| Ljubljana | 1 (< 0.001) | 1 (< 0.001) |  | 0.68 | 74 | 0.11 |
| Castile and León | 3.85 (0.003) |  | 1 (< 0.001) | 0.74 | 81 | 0.71 |
| North | 1.84 (< 0.001) |  | 1 (< 0.001) | 0.91 | 93 | 0.93 |
| Haifa | 1.87 (< 0.001) | 1.66 (< 0.001) | 1 (0.09) | 0.86 | 87 | 0.84 |
| Center | 1 (<0.001) | 1.91 (< 0.001) |  | 0.95 | 96 | 0.98 |
| Tel Aviv | 1 (< 0.001) | 1.81 (0.01) |  | 0.95 | 94 | 0.97 |
| Jerusalem | 1 (< 0.001) | 1.48 (0.09) | 1 (< 0.001) | 0.84 | 87 | 0.96 |
| South | 1 (< 0.001) | 1 (0.04) | 1.6 (< 0.001) | 0.95 | 96 | 0.97 |

* EDF is the effective degree of freedom for the estimated smooth terms. Meteorological parameter units: °C for minimum temperature, mm/day for precipitation, W/m^2^ for solar radiation.

^ǂ^ Correlation coefficient between the estimated influenza-associated ILI or ARI with the observed during 2010/2011 season
