# Supplementary material for: Associations between Meteorological Parameters and Influenza Activity in Berlin (Germany), Ljubljana (Slovenia), Castile and León (Spain) and Israeli Districts
Source: PLoS One. 2015 Aug 26;10(8):e0134701. doi: 10.1371/journal.pone.0134701 (PMC4550247; doi:10.1371/journal.pone.0134701)
Supplement: S2 Table — (DOCX) [file pone.0134701.s013.docx]

S2 Table. Regression model for excess ILI or ARI (Model 2 with minimum temperature).

| **Location** | **Meteorological Smooth Terms EDF (p-value)*** | | | **Adj. R^2^** | **% Dev. Explained** | | **Pred. Corr. Coeff.**^ǂ^ |
| --- | --- | --- | --- | --- | --- | --- | --- |
|  | **Minimum Temperature** | **Precipitation** | **Solar Radiation** |  |  |  |  |
| Berlin | 1 (0.003) |  |  | 0.50 | 42 | 0.60 | |
| Ljubljana | 1.73 (< 0.001) | 1 (< 0.001) |  | 0.68 | 74 | 0.11 | |
| Castile and León | 4.57 (0.007) |  | 1 (< 0.001) | 0.76 | 82 | 0.72 | |
| North | 1 (< 0.001) | 2.86 (< 0.001) | 2.93 (< 0.001) | 0.94 | 93 | 0.87 | |
| Haifa | 1.81 (< 0.001) |  |  | 0.75 | 85 | 0.85 | |
| Center | 1.69 (< 0.001) | 1.9 (0.001) |  | 0.96 | 96 | 0.95 | |
| Tel Aviv | 1 (< 0.001) | 1.77 (0.06) |  | 0.94 | 94 | 0.93 | |
| Jerusalem | 1 (< 0.001) | 1.6 (0.005) | 1 (0.03) | 0.88 | 88 | 0.96 | |
| South | 1 (< 0.001) |  | 1.79 (0.04) | 0.96 | 96 | 0.97 | |

* EDF is the effective degree of freedom for the estimated smooth terms. Meteorological parameter units: °C for minimum temperature, mm/day for precipitation, W/m^2^ for solar radiation.

^ǂ^ Correlation coefficient between the estimated influenza-associated ILI or ARI with the observed during 2010/2011 season
