# Supplementary material for: Associations between Meteorological Parameters and Influenza Activity in Berlin (Germany), Ljubljana (Slovenia), Castile and León (Spain) and Israeli Districts
Source: PLoS One. 2015 Aug 26;10(8):e0134701. doi: 10.1371/journal.pone.0134701 (PMC4550247; doi:10.1371/journal.pone.0134701)
Supplement: S1 Text — (DOCX) [file pone.0134701.s014.docx]

S1 TEXT

**A. Influenza Sentinel Surveillance System**

*Germany*

Acute Respiratory Infection (ARI) syndromic sentinel surveillance in Germany is based on voluntarily participating private physicians in primary care. ARI case patients are defined as patients with acute pharyngitis, bronchitis or pneumonia, with or without fever. A subset of sentinel physicians also performs virological surveillance for influenza. Each week, those physicians take 3 to 5 samples from the upper respiratory tract of influenza like illness (ILI) case patients, which are defined as persons with fever and either cough or sore throat. Physicians send these samples to the National Reference Center for Influenza (NRCI)[1]. Data is collected year round.

*Israel*

The Israel Center for Disease Control monitors influenza activity using a population-based surveillance system that consists of clinical and virological surveillance. For clinical surveillance, morbidity data is collected throughout the year from patient visits to community clinics that are part of the second largest health maintenance organizations, which serves about 25% of Israeli population. Patients diagnosed with influenza based on clinical symptoms are referred to as cases of influenza-like illness. Virological surveillance is based on a network of approximately 20 sentinel clinics throughout the country. Nasopharyngeal swabs are taken during winter from patients presenting ILI symptoms. ILI is defined as fever (>37.8°C) accompanied with one of the following symptoms: cough, sore throat, runny nose, headache, general weakness, myalgia, shivering or stomach ache. Each clinic collects about 10 samples per week. All data is collected from week 40 in each year to week 16 of the following year. In this study, we included 6 Israeli districts: North, Haifa, Center, Tel Aviv, Jerusalem and South.

*Slovenia*

Influenza is monitored through a national sentinel network of 45 out-patient practices which provide care for 4% of Slovenian population. The network practices (public or private) are located all over the country to provide geographical representativeness. The physicians report weekly numbers of cases of ILI and ARI during the whole year. The network in the township of Ljubljana, Slovenia’s capital, consists of 15 primary care physicians (6 general practitioners, 3 pediatricians and 6 school doctors). The respiratory samples are taken from ILI case-patients only; preferably in first 3 days of illness. ILI is defined as sudden onset of high fever, malaise, myalgia and at least one of the following respiratory symptoms: cough, coryza or sore throat.

While epidemiological data are collected throughout of the year, most of the virological samples are taken during winter months.

*Spain*

Influenza data for Castile and León, one of the autonomous communities in Spain, is collected through the Health Sentinel Network (HSN). Between 30 and 45 general practitioners (GP) and pediatricians from primary care participate in the HSN and collect clinical and epidemiological data from ILI patients. These primary care clinicians have been selected randomly in order to have a representative population [2]. Between 2000 to 2008/09 the International Classification of Health Problems in Primary Care (ICHPCC-2) [3] ILI case definition was used: presence of at least four out of the following eight criteria – fever, sudden onset, cough, inflammation of nasal mucous membrane and throat, chills, asthenia, myalgia and contact with an influenza patient. From 2009/10 onwards, the EU definition for ILI case was applied [4]: sudden onset of symptoms, one of the four systemic symptoms (fever, malaise, headache, myalgia) and one of the three respiratory symptoms (cough, sore throat, shortness of breath).The sentinel clinicians collect specimens during the season with informed consent of the patient and when the time between the first symptom and the swab is not more than 72 hours. Data is collected each year from week 40 to week 20 of the following year.

**B. Analytical Approach**

Generalized additive model (GAM) was used with cubic smoothing spline of the meteorological variable. Briefly, a cubic spline is constructed using sections of cubic polynomial curve joined together at their “knots”, which are evenly located through the range of the meteorological variable [5]. Each section of the cubic polynomial has different coefficients; hence a GAM model with one cubic spline smooth term may have more than one coefficient. For any meteorological variable *x*, the smooth spline function *s(x)* can generally be written as:

$$s(x) = \sum_{j=1}^{q} b_{j}(x)\beta_{j}$$

where *β_j_* are the parameters to be estimated and *b_j_(x)* are the basis functions for the cubic spline. The smooth splines were estimated by minimizing Generalized Cross Validation (GCV) which was appropriate for overdispersed Poisson [6]. The scale parameter was estimated using GCV and the Pearson estimator [5]. GCV, in general, can be written as [5]:

$$GCV = \frac{n\sum_{i=1}^{n} V(\hat{\mu}_{i})^{-1}(y_{i}-\hat{\mu}_{i})^{2}}{(n-EDF)^{2}}$$

With *n* as the number of observation; *V* the variance function; *y_i_* and *μ_i_* are the *i*-th observation and estimation, respectively; EDF is the effective degrees of freedom.

The model deviance can be written as [7]:

$$dev_{k} = 2\times\sum_{i} \ln\left(\frac{y_{ik}}{\hat{y}_{ik}}\right)-(y_{ik}-\hat{y}_{ik})$$

**C. Excess ILI and ARI calculations**

We calculated weekly excess influenza-like illness (ILI) or acute respiratory infection (ARI) cases as an indicator for influenza activity. These were the number of ILI or ARI cases that exceeded the estimated baseline values. The baseline was constructed by applying a seasonal regression model to the ILI or ARI time series outside of the influenza season. Since ILI or ARI cases may include other viral infections than influenza (such as respiratory syncytial virus), the seasonal regression was employed to estimate illnesses caused by other co-circulating viruses in the absence of influenza. This approach is commonly used to estimate influenza mortality and morbidity burden in the temperate regions [8]–[12].

Since Germany used ARI data while the other countries used ILI data for influenza surveillance, we employed a different seasonal regression model for Germany. For countries other than Germany, we applied the following seasonal regression model to the weekly count of ILI outside of the influenza season:

$${ln(b}_{t,k})= \beta_{0}+\beta_{1}t + \beta_{2}\sin\left( \frac{2\pi t}{52} \right)+ \beta_{3}\cos\left( \frac{2\pi t}{52} \right)$$

Where *t* is running time index, *k* is the study location, *b_t,k_* is the estimated baseline value at time *t* and location *k*, and *β_0_* to *β_3_* are the estimated intercept and regression coefficients.

For Germany, we followed the method used by Germany’s Robert Koch Institute [1] to construct the baseline. This method was similar to the one shown above, but with additional sinusoidal functions with higher frequencies. We fitted the following seasonal regression model to the weekly count of ARI outside the influenza season:

$${ln(b}_{t,k})= \beta_{0}+\sum_{i=1}^{M} {\beta_{1i}t}^{i}+ \sum_{j=2}^{N} \left( \beta_{2j}\sin\left( \frac{2\pi tj}{52} \right)+ \beta_{3j}\cos\left( \frac{2\pi tj}{52} \right) \right)+ \sum_{l=2}^{P} \left( \beta_{4l}\sin\left( \frac{2\pi t}{l \times52} \right)+ \beta_{5l}\cos\left( \frac{2\pi t}{l \times52} \right) \right)$$

Here, we used M = 3, N = 6 and P = 3 as suggested in [1].

Each of the 4 countries has its own definition of influenza season. In Spain, the start of influenza season was defined as the week in which the ILI rate was greater than the epidemic threshold calculated, every season, by the Moving Epidemic Method [13], [14]. In Israel, the start and end of influenza season were defined as the first and the last of two consecutive weeks with nasopharyngeal swabs positive to influenza, respectively (weeks in which there were less than 10 samples collected were considered as weeks with no influenza activity). In Slovenia, the season was defined as the weeks in which ILI rate was greater than 25/100,000 population with proportion of samples tested positive for influenza ≥ 30%. In Germany, the start and end of an epidemic period was identified as the first two consecutive weeks with the lower 95%-confidence limit of the positivity rate ≥ 10% (for the start) and <10% (for the end) [1].

The excess ILI or ARI was then calculated as the amount of ILI or ARI that exceeded the upper 95% confidence limit of the estimated baseline *b_t,k_*.

$$z_{t,k}= y_{t,k}-b_{t,k}^{UL}$$

Here, *z_t,k_* is the excess ILI or ARI, *y_t,k_* is the observed ILI or ARI, and $b_{t,k}^{UL}$ is the estimated upper 95% confidence limit of the baseline value, with *t* and *k* indicate time and location respectively. We further expressed *z_t,k_* as excess ILI or ARI per 100,000 population by dividing it with the number of population. The estimated baselines are shown in S3 Fig.

**Results Using Excess ILI and ARI**

*Specific Humidity Model*

Similar to the results presented in the main text, we found that specific humidity was associated with excess ILI or ARI in all locations (S1 Table), although in this case most locations indicated a nonlinear relationship and generally with a downward trend (S4 Fig.). When specific humidity was approximately below the median value, there were significant inverse associations with excess ILI. In Castile and León, there was a slight upward trend between specific humidity and excess ILI at very low and intermediate values of specific humidity Such second or higher order variations with specific humidity were also observed in some Central American countries [Soebiyanto & Kiang, personal communication].

The association between excess ILI or ARI with precipitation was found in 1 temperate location (Ljubljana) and 4 out of the 6 Israeli districts, and the directions of the association were inconclusive. Some locations showed a downward trend (inverse association) between precipitation and excess ILI or ARI (Ljubljana and South); upward trend or proportional association (Haifa, Center and Tel Aviv Solar radiation was significantly associated with excess ILI or ARI in 4 locations (Castile and León, North, Jerusalem and South). In these locations, the association between them showed a downward trend (inverse association)

The specific humidity models accounted 42% to 96% of the deviance, with R^2^ ranging from 0.49 to 0.95 (S1 Table). When the models were used to estimate the 2010/2011 seasons, we found that the models closely followed the observed excess ILI or ARI in all locations except in Ljubljana (S5 Fig.). In these locations the correlation coefficient between the estimate and observation ranged from 0.56 (in Berlin) to 0.98 (in Center). In Haifa, the models underestimated excess ILI or ARI around the peak week. In Ljubljana the models could not estimate the 2010/2011 season accurately (correlation coefficient of 0.11). These results were similar to what we reported in the main text.

*Minimum Temperature Model*

Using minimum temperature model, we found that all locations were associated with minimum temperature, while the association with precipitation and solar radiation remained location-dependent (S2 Table). In Castile and León, excess ILI or ARI demonstrated a highly nonlinear association with minimum temperature (S6 Fig.). In the other locations (Berlin, Ljubljana, North, Haifa, Tel Aviv and South), minimum temperature showed an inverse association with excess ILI or ARI.

Similar to the specific humidity models, the association between precipitation and excess ILI or ARI were inconclusive (S6 Fig.), with some locations showing a downward trend (Ljubljana, North and Jerusalem) and an upward trend in Center district. Meanwhile, excess ILI or ARI showed an inverse association with solar radiation in 3 locations (Castile and León, Jerusalem and South) and both upward and downward trend in North district (S6 Fig.).

The adjusted R^2^ values for minimum temperature models ranged from 0.5 to .0.96 with 42% to 96% deviance explained. Similar to the specific humidity models, when the minimum temperature models were used to estimate the excess ILI or ARI in 2010/2011 season, the estimated values closely followed the observations in all locations except in Ljubljana (S7 Fig.). This was also reflected in the low correlation coefficients between the estimated and observed excess ILI in Ljubljana which was the lowest (0.11). In other locations, the correlation coefficients ranged from 0.56 to 0.98 (S2 Table).

**References**

[1] M. A. der Heiden, K. Köpke, S. Buda, U. Buchholz, and W. Haas, “Estimates of excess medically attended acute respiratory infections in periods of seasonal and pandemic influenza in Germany from 2001/02 to 2010/11.,” *PLoS One*, vol. 8, no. 7, p. e64593, Jan. 2013.

[2] A. . Vega Alonso, M. Gil, C. Cosín, and E. Zapatero, “La Red de Médicos Centinelas de Castilla y León: Aplicación del análisis de conglomerados para la obtención de una muestra representativa,” *Gac. Sanit.*, vol. 20, no. 4, pp. 184–188, 1990.

[3] World Organization of National Colleges of Physician, *International Classification of Health Problems in Primary Care (ICHPPC-2-Defined)*, Edición Es. Barcelona: Masson CA, 1988.

[4] European Centre for Disease Prevention and Control, “Influenza Case Definitions,” 2013. [Online]. Available: http://ecdc.europa.eu/en/activities/surveillance/eisn/surveillance/pages/influenza_case_definitions.aspx. [Accessed: 14-Mar-2013].

[5] S. Wood, *Generalized Additive Models: An Introduction with R*. Chapman and Hall/CRC, 2006, p. 410.

[6] S. Wood, “mgcv: GAMs and Generalized Ridge Regression for R,” *R News*, vol. 1, no. 2, pp. 20–25, 2001.

[7] M. J. Crawley, *The R Book*. West Sussex, England: John Wiley & Sons, Ltd, 2007, p. 942.

[8] G. Chowell, M. A. Miller, and C. Viboud, “Seasonal influenza in the United States, France, and Australia: transmission and prospects for control.,” *Epidemiol. Infect.*, vol. 136, no. 6, pp. 852–64, Jun. 2008.

[9] J. Shaman, V. E. Pitzer, C. Viboud, B. T. Grenfell, and M. Lipsitch, “Absolute humidity and the seasonal onset of influenza in the continental United States.,” *PLoS Biol.*, vol. 8, no. 2, p. e1000316, Feb. 2010.

[10] W. W. Thompson, L. Comanor, and D. K. Shay, “Epidemiology of seasonal influenza: use of surveillance data and statistical models to estimate the burden of disease.,” *J. Infect. Dis.*, vol. 194 Suppl , pp. S82–91, Nov. 2006.

[11] W. W. Thompson, D. K. Shay, E. Weintraub, L. Brammer, C. B. Bridges, N. J. Cox, and K. Fukuda, “Influenza-associated hospitalizations in the United States,” *JAMA*, vol. 292, no. 11, pp. 1333 – 1340, 2004.

[12] C.-M. Liao, S.-Y. Chang, S.-C. Chen, and C.-P. Chio, “Influenza-associated morbidity in subtropical Taiwan,” *Int. J. Infect. Dis.*, vol. 13, pp. 589 – 599, 2009.

[13] T. Vega, J. E. Lozano, T. Meerhoff, R. Snacken, J. Mott, R. Ortiz de Lejarazu, and B. Nunes, “Influenza surveillance in Europe: establishing epidemic thresholds by the moving epidemic method.,” *Influenza Other Respi. Viruses*, vol. 7, no. 4, pp. 546–58, Jul. 2013.

[14] T. Vega Alonso, J. E. Lozano Alonso, R. Ortiz de Lejarazu, and M. Gutiérrez Pérez, “Modelling influenza epidemic—can we detect the beginning and predict the intensity and duration?,” *Int. Congr. Ser.*, vol. 1263, pp. 281–283, Jun. 2004.
